# Descendants of the Jurassic turiasaurs from Iberia found refuge in the Early Cretaceous of western USA

**DOI:** 10.1038/s41598-017-14677-2

**Published:** 2017-10-30

**Authors:** Rafael Royo-Torres, Paul Upchurch, James I. Kirkland, Donald D. DeBlieux, John R. Foster, Alberto Cobos, Luis Alcalá

**Affiliations:** 1 Fundación Conjunto Paleontológico de Teruel-Dinópolis/Museo Aragonés de Paleontología, Av. Sagunto s/n, E-44002 Teruel, Spain; 2 Department of Earth Sciences, University College London, Gower Street, London, WC1E 6BT United Kingdom; 3 Utah Geological Survey, PO Box 146100, Salt Lake City, Utah 84114-6100 United States; 4 Museum of Moab, 118 East Center St., Moab, Utah 84532 United States

## Abstract

A new, largely complete eusauropod dinosaur with cranial and postcranial elements from two skeletons, *Mierasaurus bobyoungi* gen. nov., sp. nov. from the lower Yellow Cat Member (Early Cretaceous) of Utah (USA), is the first recognized member of Turiasauria from North America. Moreover, according to our phylogenetic results, *Moabosaurus utahensis* from the lower Yellow Cat Member of Utah (USA) is also a member of this clade. This group of non-neosauropod eusauropods, which now includes five genera (*Losillasaurus*, *Turiasaurus*, *Mierasaurus*, *Moabosaurus* and *Zby*), was previously known only from the Jurassic of Europe. These recent discoveries in Utah suggest that turiasaurs as a lineage survived the Jurassic-Cretaceous extinction boundary and expanded their known range, at least, into western North America. The revised spatiotemporal distribution of turiasaurs is consistent with the presence of a land connection between North America and Europe sometime during the late Tithonian to Valanginian (*c*.147-133 Ma). *Mierasaurus* and *Moabosaurus* are the only non-neosauropod eusauropods known from North America, despite being younger than the classic neosauropods of the Morrison Formation (*c*.150 Ma).

## Introduction

**Figure 1:**
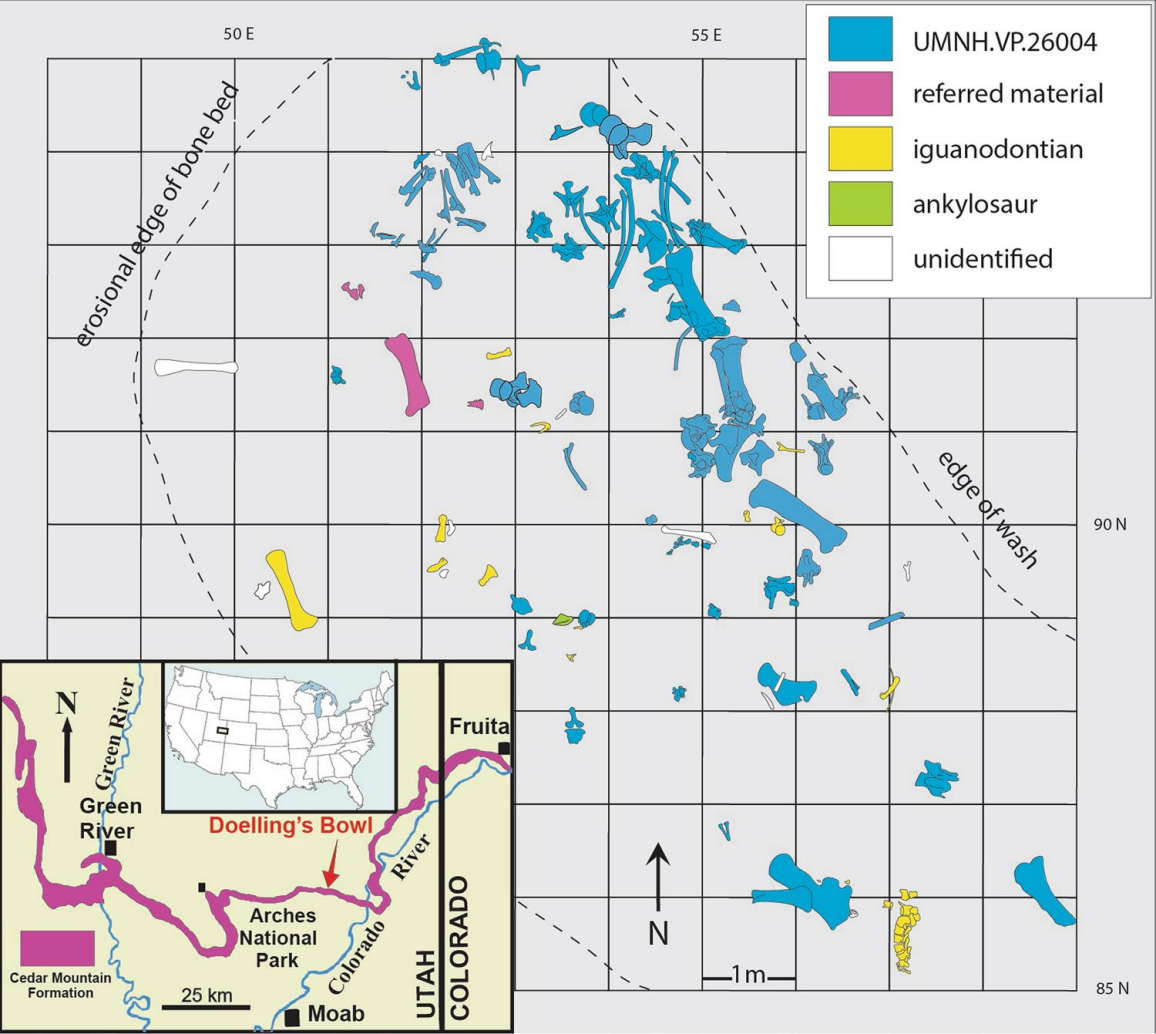
Excavation map showing the specimens of *Mierasaurus bobyoungi* gen. nov, sp. nov. and geographical setting of the Doelling’s Bowl site. Recovered specimens include: numerous disarticulated cranial elements, many axial elements, an articulated hind leg and a partial articulated forelimb (holotype: UMNH.VP.26004, and referred material of a juvenile sauropod: UMNH.VP.26010 and UMNH.VP.26011). The articulated manus and pes extended through the sediment at an angle below the level of the majority of the skeleton, indicating that an individual sauropod became mired in soft sediment and died in place. This map was drafted by J.I.K. and D.D.D. (© Utah Geological Survey) in Adobe Illustrator CS5 (www.adobe.com/es/products/illustrator.html).

**Figure 2:**
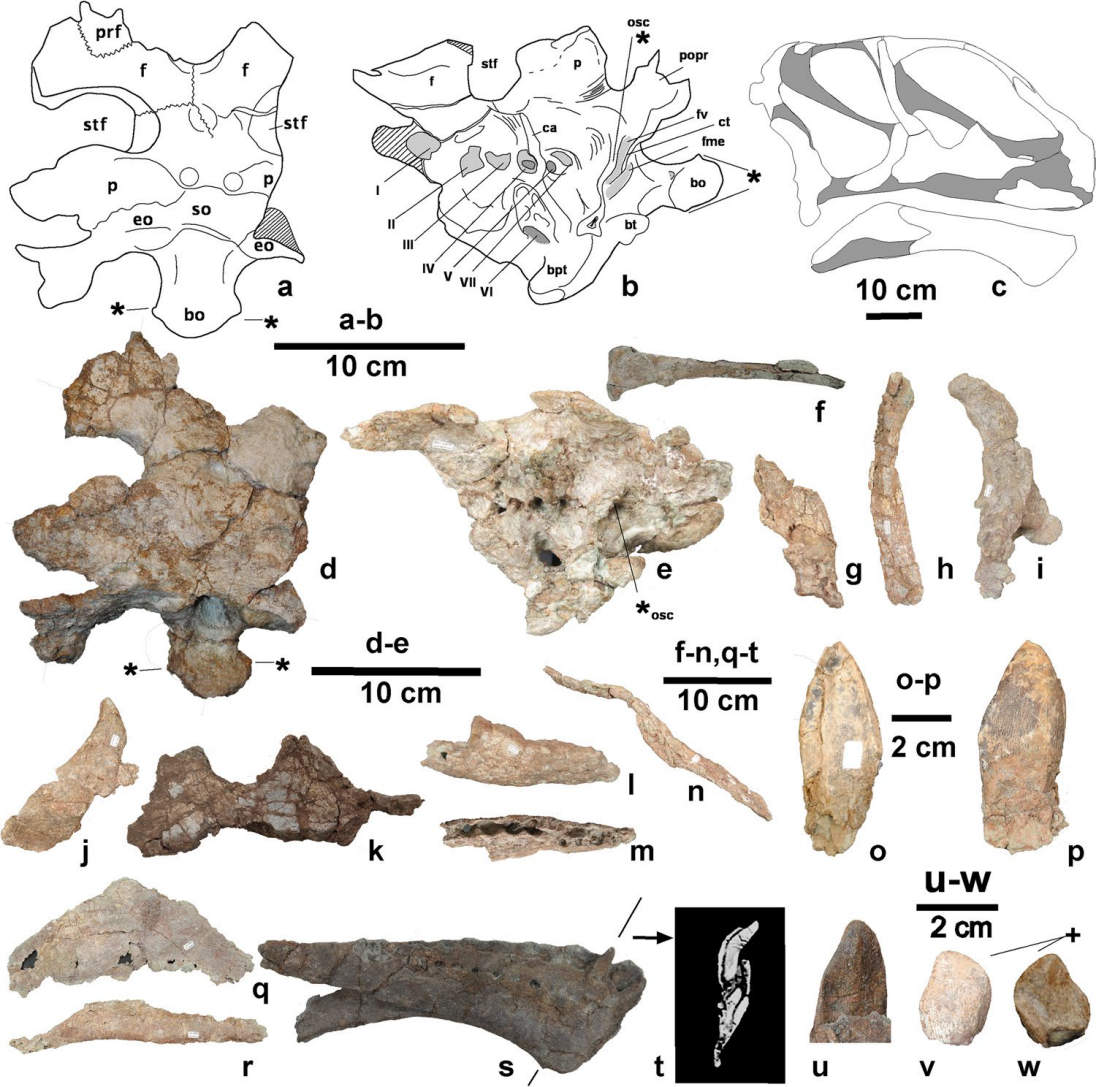
The skull material (UMNH.VP.26004) of *Mierasaurus bobyoungi* gen. nov, sp. nov.: (a) braincase (DBGI 173) in dorsal view; (b) braincase (DBGI 173) in left ventrolateral view; (c) reconstruction of the skull of *Mierasaurus*. Shaded bones were either not preserved or yielded no information if they were; (e) braincase (DBGI 173) in left lateral view; (f) nasal (DBGI 85 A) in dorsal view; (g) anterior fragment (muzzle) of the right premaxilla (DBGI 107 D) in left lateral view; (h) right lacrimal (DBGI 160) in left lateral view; (i) right quadrate (DBGI 54) in right lateral view; (j) right jugal (DBGI 78B) in right lateral view; (k) fragment of right maxilla (DBGI 78) in left lateral view (l) fragment of the right maxilla (DBGI 95I) in left lateral view; (m) fragment of the right maxilla (DBGI 95I) in ventral view; (n) fragment of nasal and ascending process of the premaxilla (DBGI 107D) in right lateral view; (o) a premaxillary-maxillary tooth (DBGI 95) in lingual view; (p) a premaxillary-maxillary tooth (DBGI 95) in labial view; (q) right surangular (DBGI 71) in right lateral view; (r) left prearticular (DBGI 70) in lateral view; (s) left dentary (DBGI 60) in medial view; (t) CT image of the dentary in transverse cross-section showing two replacement teeth; (u) anterior dentary teeth (DBGI 60) in lingual view; (v) posterior tooth (DBGI 27) in labial view; (w) posterior tooth (DBGI 27) in lingual view. A plus sign (+) indicates a synapomorphy of Turiasauria: teeth with a heart-shaped outline. An asterisk (*) indicates autapomorhies of *Mierasaurus bobyoungi* gen. et sp. nov. For abbreviations see supplementary information. (a), (b) and (c) were drafted by R.R.T. (© Fundación Conjunto Paleontológico de Teruel-Dinópolis) in Adobe Illustrator CS5 (www.adobe.com/es/products/illustrator.html).

**Figure 3:**
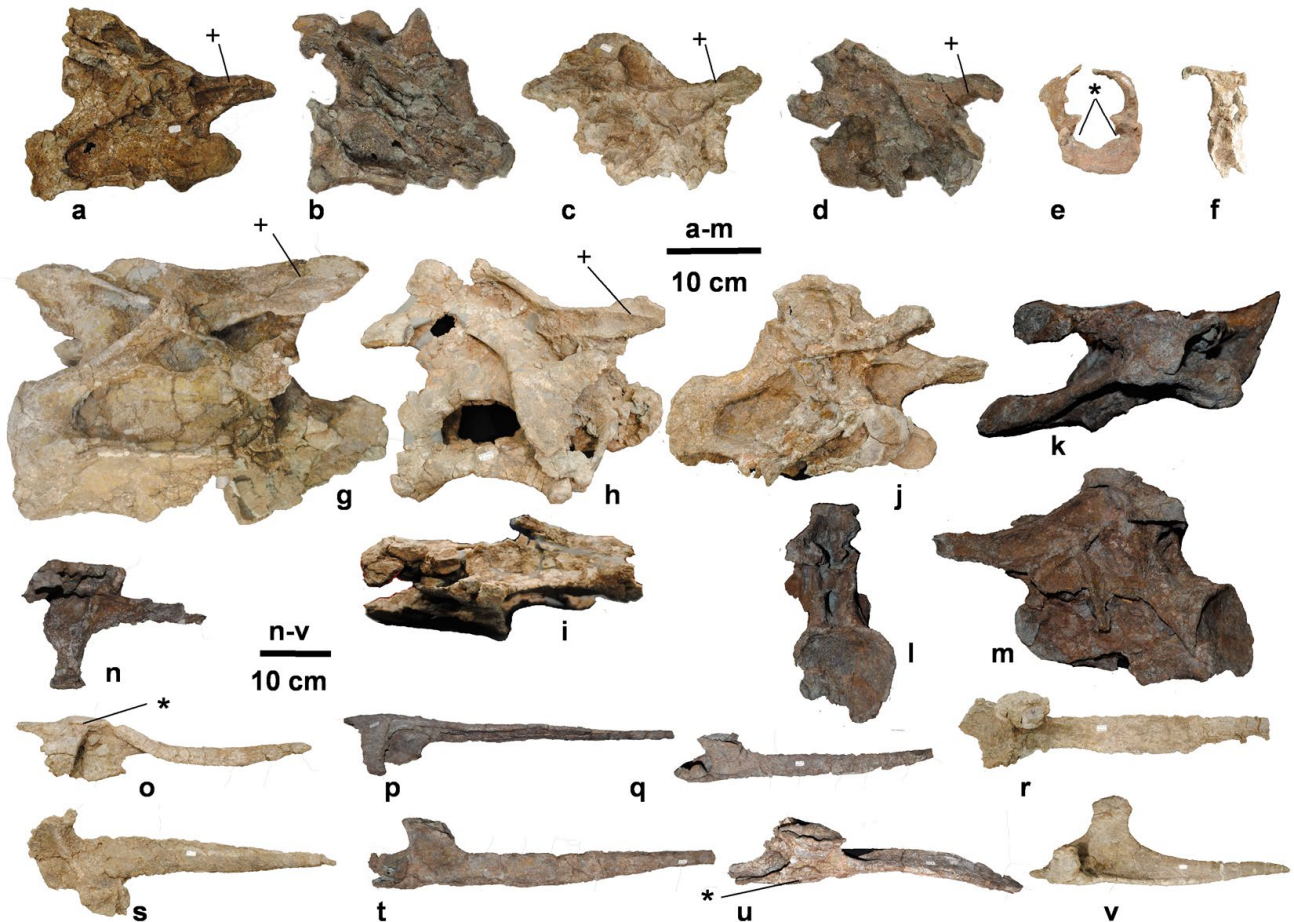
The postcranial skeleton (UMNH.VP.26004) of *Mierasaurus bobyoungi* gen. nov, sp. nov. with the following elements: (a) middle cervical vertebra (DBGI 69 h) in right lateral view; (b) middle cervical vertebra (DBGI 69G1) in right lateral view; (c) anterior cervical vertebra (DBGI 165) in right lateral view; (d) anterior cervical vertebra (DBGI 69G2) in right lateral view; (e) atlas (DBGI 5I) in anterior view; (f) atlas (DBGI 5I) in right lateral view; (g) posterior cervical vertebra (DBGI 95) in right lateral view; (h) posterior cervical vertebra (DBGI 19 A) in right lateral view; (i) posterior cervical vertebra (DBGI 19 A) in ventral view; (j) middle cervical vertebra (DBGI 38) in right lateral view; (k) middle cervical vertebra (DBGI 38) in dorsal view; (l) middle cervical vertebra in posterior view; (m) middle cervical vertebra (DBGI 38) in left lateral view; (n) right anterior cervical rib (DBGI 5D) in medial view; (o) right anterior cervical rib (DBGI 28 A) in medial view; (p) right anterior-middle cervical rib (DBGI 95 C) in medial view; (q) right middle cervical rib (DBGI 45 F) in dorsal view; (r) right middle cervical rib (DBGI 95 A) in dorsal view; (s) left anterior cervical rib (DBGI 95B) in lateral view; (t) left middle cervical rib (DBGI 95 H) in lateral view; (u) left middle cervical rib (DBGI 95D) in dorsal view; (v) right posterior cervical rib (DBGI 10) in dorsal view. A plus sign (+) indicates a diagnostic character for *Mierasaurus bobyoungi* gen. et sp. nov. An asterisk (*) indicates an autapomorphy of *Mierasaurus bobyoungi* gen. et sp. nov. (© Fundación Conjunto Paleontológico de Teruel-Dinópolis) in Adobe Illustrator CS5 (www.adobe.com/es/products/illustrator.html).

**Figure 4:**
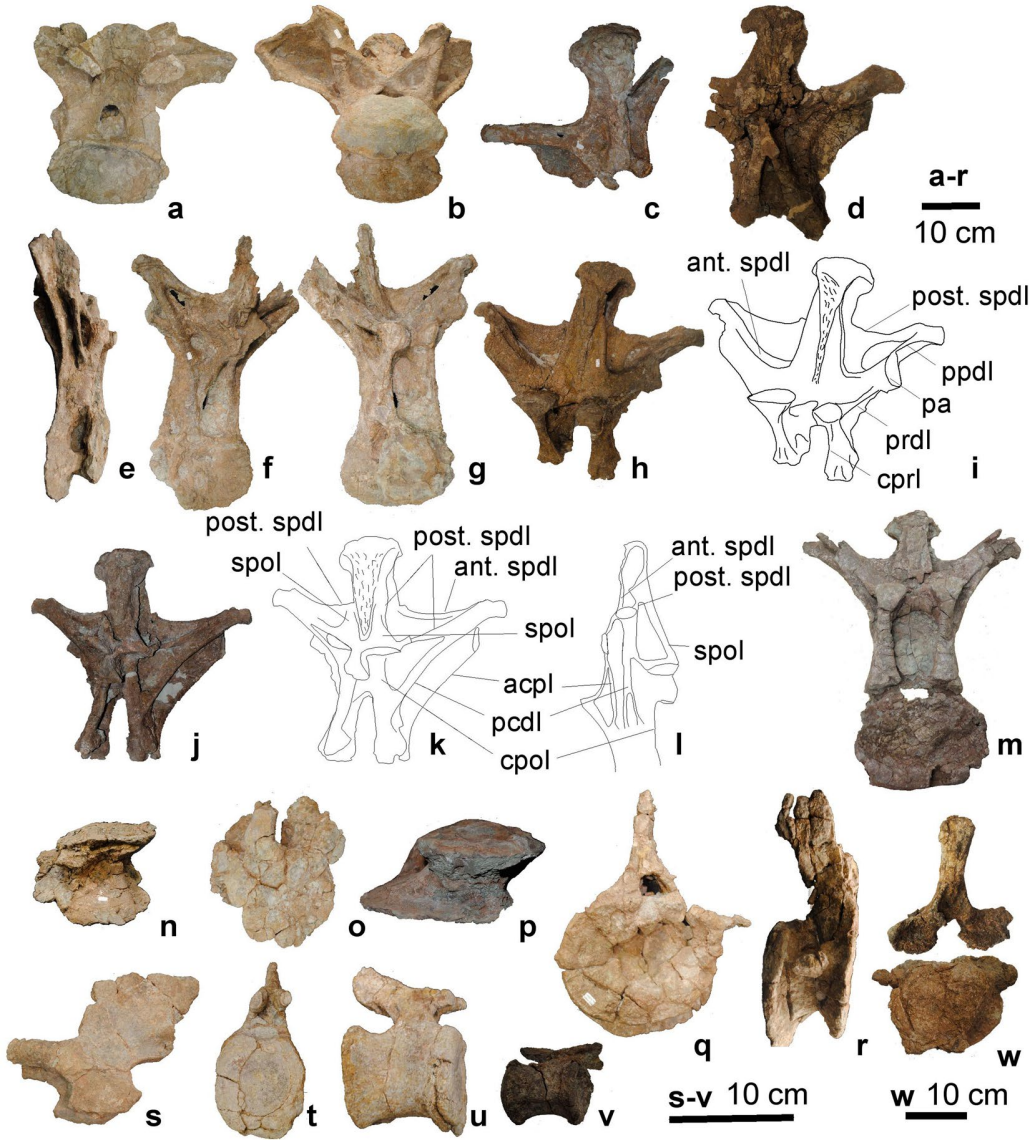
The postcranial skeleton (UMNH.VP.26004) of *Mierasaurus bobyoungi* gen. nov, sp. nov. with the following elements: (a) anterior dorsal vertebra (DBGI 54 A) in posterior view; (b) anterior dorsal vertebra (DBGI 54 A) in anteroventral view; (c) neural arch of a middle dorsal vertebra (DBGI 37) in right anterolateral view; (d) posterior neural arch of a dorsal vertebra (DBGI 19 A) in posterior view; (e) anterior dorsal vertebra (DBGI 16) in right lateral view; (f) anterior dorsal vertebra (DBGI 16) in posterior view; (g) posterior dorsal vertebra (DBGI 16) in anterior view; (h,i) posterior dorsal vertebra (DBGI 100NA 1) in anterior view; (j,k) posterior dorsal vertebra (DBGI 100NA 1) in posterior view; (l) posterior dorsal vertebra (DBGI 100NA 1) in left lateral view; (m) middle dorsal vertebra (DBGI 11) in anterior view; (n) centrum of a posterior dorsal vertebra (DBGI 24B) in ventral view; (o) centrum of a posterior dorsal vertebra (DBGI 24B) in anterior view; (p) centrum of a posterior dorsal vertebra (DBGI 192) in ventral view; (q) anterior-middle caudal vertebra (DBGI 23B) in anterior view; (r) anterior-middle caudal vertebra (DBGI 23B) in right lateral view; (s) posterior neural arch of a posterior caudal vertebra (DBGI 48) in left lateral view; (t) posterior caudal vertebra (DBGI 21) in anterior view; (u) posterior caudal vertebra (DBGI 21) in right lateral view; (v) distal caudal vertebra (DBI 37-34-529) in right lateral view; (W) anterior caudal vertebra (DBGI 192) in posterior view. For abbreviations see supplementary information. (i), (k) and (l) were drafted by R.R.T. (© Fundación Conjunto Paleontológico de Teruel-Dinópolis) in Adobe Illustrator CS5 (www.adobe.com/es/products/illustrator.html).

**Figure 5:**
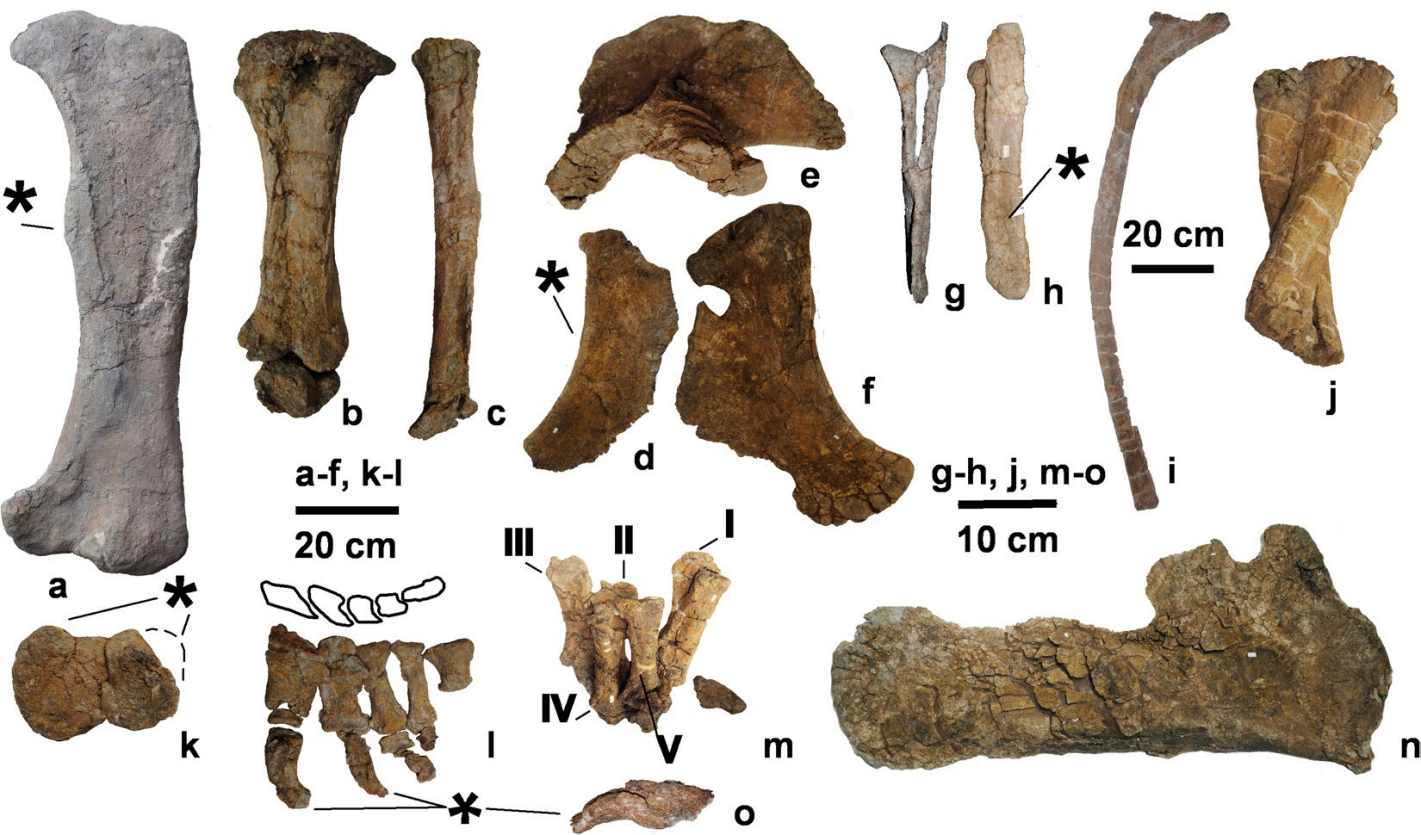
The postcranial skeleton (UMNH.VP.26004) of *Mierasaurus bobyoungi* gen. nov, sp. nov. with the following elements: (a) right femur (DBGI 39) in posterior view; (b) left tibia and astragalus (DBGI 75) in posterior view; (c) left fibula (DBGI 75) in posterior view; (d) right ischium (DBGI 69 A) in lateral view; (e) left ilium (DBGI 100IL) in medial view; (f) right pubis (DBGI 195) in lateral view; (g) anterior/middle chevron (DBGI 172) in posterior view; (h) anterior/middle chevron (DBGI 172) in right lateral view; (i) dorsal rib (DBGI 100 R1) in medial view; (j) left articulated ulna and radius (DBGI 100R) in anterior view; (k) left femur (DBGI 39) in distal view; (l) a complete left pes (DBGI 75) in dorsal view; (m) left articulated manus (DBGI 100 M) in anterior view; (n) right scapula (DBGI 250) in lateral view; (o) left pedal ungual II in medial view. An asterisk (*) indicates an autapomorphy of *Mierasaurus bobyoungi* gen. et sp. nov. (l) was drafted by R.R.T. (© Fundación Conjunto Paleontológico de Teruel-Dinópolis) in Adobe Illustrator CS5 (www.adobe.com/es/products/illustrator.html).

**Figure 6:**
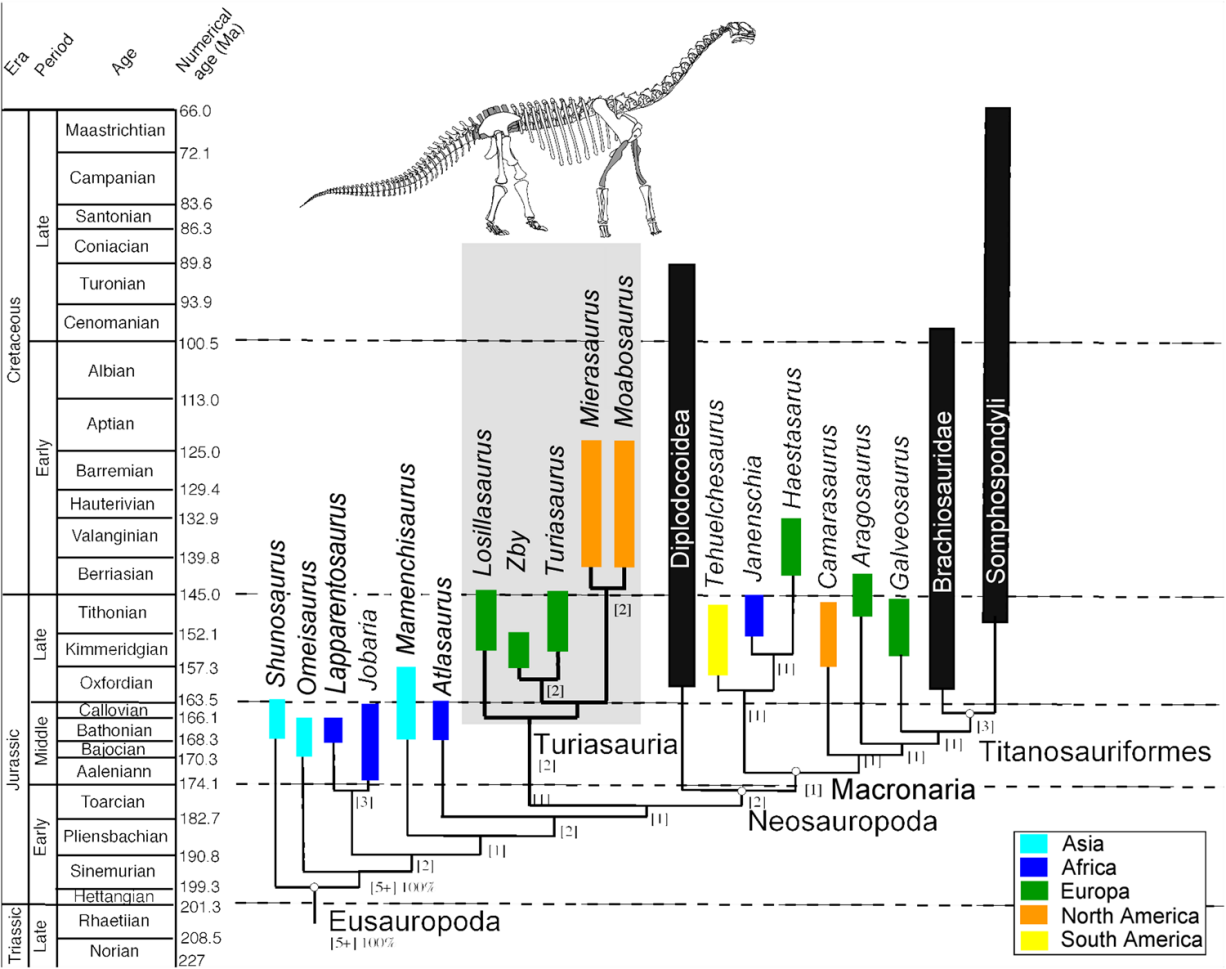
Phylogenetic relationships of Turiasauria: The time-calibrated^65^ phylogenetic relationships (see Supplementary information and Supplementary Fig. S5) of *Mierasaurus bobyoungi* n. gen. et sp. nov. The box next to each taxon demarcates its temporal range, whereas the colour of the box reflects the continent(s) where the taxon occurs (yellow = South America, light blue = Asia, orange = North America; green = Europe, dark blue = Africa; black = several continents). This figure was drafted by R.R.T. in Adobe Illustrator CS5 (www.adobe.com/es/products/illustrator.html) © Fundación Conjunto Paleontológico de Teruel-Dinópolis.

Sauropod dinosaurs were the largest vertebrates ever to walk the Earth^1,2^ and were a diverse and successful group^3^. Although they had achieved a global distribution by the Middle Jurassic and maintained their dominance until the end of the Cretaceous^3,4^, their evolutionary history was not without its crises. In particular, they suffered a 60-80% extinction at the end of the Jurassic^3–7^, which was part of a much wider extinction event affecting many marine and terrestrial groups^7,8^. This apparently resulted in the extinction of most of the non-neosauropod eusauropods that had dominated Jurassic faunas, and their replacement by neosauropods in the Cretaceous^3,4,7^. However, recent work has suggested that lower diversity at this time was not the result of a true mass extinction occurring at the Jurassic-Cretaceous boundary itself; rather, it represents a sequence of turnover events that lasted well into the Early Cretaceous^6,7^. The non-neosauropod eusauropod lineage Turiasauria, previously only known from the Late Jurassic of Europe^1,9,10^ and possibly the Middle Jurassic of Africa^11^, was therefore a potential victim of an extinction at the Jurassic-Cretaceous boundary^4,5,7^. However, here we report the unexpected occurrence of a new turiasaur from North America, dating to the late Berriasian-early Aptian (c.142-124 Ma) a time interval in which sauropod remains are rare globally^5,12^. This discovery supports a more complex pattern of turnover among sauropods during the Jurassic-Cretaceous transition and is consistent with previously proposed palaeobiogeographic and palaeogeographic connections between Europe and North America after the Tithonian^13–16^. Thus, during the late Tithonian-early Aptian (*c*.147-124 Ma), non-neosauropod turiasaurs were unexpectedly present, at least, in the Utah region of North America. Importantly, this relatively rare group of sauropods are represented in North America by a new taxon described here as *Mierasaurus bobyoungi* gen. nov, sp. nov. (Figs 1–5), which is closely related to the Kimmeridgian-Tithonian turiasaurs (*Losillasaurus*, *Turiasaurus* and *Zby*) of Europe^1,9,10^ and the recently published Early Cretaceous taxon (from the lower Yellow Cat Member) *Moabosaurus utahensis* ^17^, after the revised phylogenetic placement of the latter proposed in this work (Fig. 6 and Supplementary Figs S4 and S5).

## Results

### Systematic Palaeontology

Dinosauria Owen, 1842

Saurischia Seeley, 1887

Sauropoda Marsh, 1878

Eusauropoda Upchurch, 1995

Turiasauria Royo-Torres, Cobos and Alcalá, 2006

***Mierasaurus bobyoungi*** gen. nov., sp. nov.

### Etymology

Genus named for Bernardo de Miera y Pacheco, Spanish cartographer and chief scientist for the 1776 Domínguez-Escalante Expedition: the first European scientist to enter what is now Utah. The species name acknowledges the importance of the underappreciated research by Robert Young on the Early Cretaceous of Utah^18^.

### Holotype

A partial skeleton of a single individual (UMNH.VP.26004), comprising disarticulated cranial and postcranial elements from the type site (Doelling’s Bowl). We regard this as subadult because it is a relatively large animal with unfused vertebral centra and neural arches in some dorsal vertebrae. This individual includes a partial skull and jaw, teeth, atlas, 8 cervical vertebrae, 11 cervical ribs, 11 dorsal vertebrae, 6 dorsal ribs, 6 sacral ribs, 15 caudal vertebrae, two chevrons, right scapula and partial left scapula, left radius, left ulna, left manus, complete pelvic elements, both femora, left tibia, left fibula, left astragalus and left pes (Figs 1–5; Supplementary Tables S1 and S2 and Supplementary Fig. S6).

### Referred material

Other disarticulated sauropod specimens from the type locality include a possible juvenile dentary (UMNH.VP.26010) and juvenile femur (UMNH.VP.26011) (Fig. 1).

### Type locality and horizon

All *Mierasaurus* remains discussed herein are from Doelling’s Bowl bonebed (Fig. 1 and Supplementary Figs S1-S3), UMNH VP.LOC.1208 (Utah Loc. 42Gr0300v) within the lower Yellow Cat Member (below the marker calcrete), Cedar Mountain Formation, on lands managed by the U.S. Bureau of Land Management in northern Grand County, east-central Utah. In a preliminary report in a conference abstract, detrital zircon dating indicates maximum ages ranging from ~136.4 ± 1.1 Ma and ~132 Ma for lower Yellow Cat Member^19^ and ~137.2 ± 2.0 Ma for upper Yellow Cat Member^19^ (Supplementary Figs S1-S3). The combination of these data with the upper Berriasian-Valanginian age based on ostracods^20^ and charophyte fauna^21^ for the upper Yellow Cat Member indicates a conflict in the maximum age traditionally considered for it 124.2 ± 2.6 Ma^17,22^ (see Supplementary Information). All of these data suggest a potential age of late Berriasian-early Aptian (*c*.142-124 Ma) for the Yellow Cat Member.

Doelling’s Bowl has produced the iguanodont cf. *Iguanocolossus* sp., a new species of polacanthid ankylosaur, a large allosauroid theropod (teeth), and the dromaeosaur *Yurgovuchia doellingi* ^23–25^. Exact locality information will be provided to qualified researchers on request through the Natural History Museum of Utah or the Utah Geological Survey.

### Diagnosis

A turiasaurian sauropod possessing the following features (autapomorphies marked by *): *the otosphenoidal ridge extends from the anterior surface of the paroccipital process, near its ventral margin and is restricted to the medialmost part of the latter process (Fig. 2b); *the occipital condyle has a pair of rounded ridges extending dorsoventrally, one on either lateral face of the condylar articular surface (Fig. 2a–d); *the atlantal intercentrum (Fig. 3e,f) bears a pair of depressions in the medial surface, facing posteromedially, each of which receives the anterolateral margin of the odontoid process; a well-developed spinoprezygapophyseal lamina extends anteriorly (Fig. 3a–d, g–m) as a low ridge onto the lateral surface of the prezygapophyses roofing a lateral fossa on prezygapophyses in middle and posterior cervical vertebrae (shared with the diplodocine *Kaatedocus* ^26^); *cervical ribs bear a ridge or bulge on the lateral surface of the tuberculum, immediately posterior to the base of the anterior process (Fig. 3n–v); dorsal neural arches lack posterior centroparapophyseal laminae; *lateral depression on the distal ramus of haemal arches (Fig. 5g,h); metacarpal I longer than metacarpal IV, shared with Macronaria^27^ (Fig. 5m); a very short ischium compared to pubis length (ischium:pubis length ratio = 0.75) (Fig. 5d–f); the midpoint of the fourth trochanter placed in the proximal part of the femur (Fig. 5a); femur with subequal distal condyles (Fig. 5k); and pedal unguals 2 and 3 compressed dorsoventrally (Fig. 5l,o).

### Description

The braincase of *Mierasaurus* (Fig. 2a–d) resembles those of non-titanosauriform and non-diplodocoid neosauropods^3,9,27^. The frontal forms part of the anterior margin of the supratemporal fenestra (the plesiomorphic state seen in many non-sauropod sauropodomorphs, *Shunosaurus* and probably in *Turiasaurus*), whereas most frontals of other sauropods exhibit the derived condition by not participating in this fenestra^9^. The parietal dorsal surfaces are inclined such that they face slightly posteriorly, the supraoccipital faces posterodorsally, and the occipital condyle as a whole projects posteroventrally. The skull roof is essentially flat without domes or sulci. The supratemporal fenestra is large and transversely wide. The parietal extends laterally and twists to produce a vertically tall plate forming the posterior margin of the supratemporal fenestra. The squamosal is more slender and ventrally curved, and less flared and dorsally curved, than in *Camarasaurus* ^28^. The nuchal crest on the supraoccipital is very low. A pair of transversely widened proatlantal facets is present on the occiput, dorsolateral to the foramen magnum. The paroccipital process is anteroposteriorly compressed and directed laterally and moderately ventrally as in non-neosauropods^3^. The occipital condyle bears a strongly convex articular surface with two rounded ridges laterally, differing from *Turiasaurus* where the occipital condyle has small dimples and convexities^9^. A central, ventrally projecting area links the neck of the occipital condyle to the region of the basal tubera, with moderately deep grooves on either side below the otosphenoidal ridge. There appear to have been ridges extending along the lateral margins of the central, ventral projecting portions of the tubera. A clear pit is present in the basisphenoid fossa between and anterior to the basal tubera. The latter are relatively widely separated, and there is no clear anteroposteriorly thin wall linking their medial margins and partitioning the basisphenoid fossa from the basioccipital fossa (such a thin partition is present in *Moabosaurus* ^17^). The basal tubera are moderately compressed anteroposteriorly, similar to those in *Turiasaurus* ^9^, and are not sheet-like as occurs in titanosaurs and some rebbachisaurs^3,26,29^. The anterolateral portion of the tip of each basal tuber projects anteriorly, giving the free distal surface an unusual L-shaped profile in ventral view, a character state shared with the turiasaur *Moabosaurus* ^17^. The quadrate is curved such that its proximal head projects posteriorly and there is a well-developed deep posterior fossa. The dentary (based on two left elements of different size) increases in dorsoventral height towards the symphysis (Fig. 2s), thereby distinguishing it from *Jobaria* ^30^ but likening it to *Camarasaurus* ^28^ and an unpublished new turiasaur specimen (RD-28) from Riodeva (Teruel, Spain). As in flagellicaudatans, and to a lesser extent *Camarasaurus*, the ventral margin of the dentary forms a sharp, chin-like transverse ridge in lateral view^27^. There are approximately 13 alveoli in the dentary. The teeth are spatulate in labial view, and D-shaped in horizontal cross section. Each crown possesses a bulge on its labial surface between the apicobasally-directed mesial and distal grooves. There are no denticles (a possible synapomorphy uniting Turiasauria and Neosauropoda)^9,27,31^. The most posterior teeth possess the diagnostic heart-shape (Fig. 2v,w) seen in European turiasaurs^1,9,31^. The mesial-most teeth of *Mierasaurus* differ from those of *Camarasaurus* in being more pointed (Fig. 2o-p); those of the latter genus also have shorter apices and a basal bulge on the lingual face^28,31^. This kind of broad crown morphology is common in non-neosauropods, and is very different from the narrow-toothed condition seen in most neosauropods such as diplodocoids and titanosauriforms^3^.

The atlas is well preserved (Fig. 3e,f). The intercentrum is anteroposteriorly short with a wide, concave anterior face and convex posterior face. The posterolateral surface of the odontoid process fits into two posterolaterally facing depressions on the atlas intercentrum, unlike *Camarasaurus* ^28^, *Diplodocus* ^32^ or *Moabosaurus* ^17^. The atlantal neural arch bears a pair of medially directed projections that partially separate the neural canal and neurapophyses (above) from the notch in the dorsal surface of the centrum that receives the odontoid process (below). The centrum lacks the robust parapophysis described for the atlas of *Moabosaurus* ^17^.

The internal tissue structure of the presacral vertebrae and thoracic ribs is solid, lacking camerae, camellae or other pneumatic cavities: this suggests that *Mierasaurus* is not a titanosauriform sauropod^27,33,34^. The cervical vertebra neurapophyses are long curved plate-like elements that closely approach each other without actually meeting on the midline above the neural canal. The cervical centra are strongly opisthocoelous (Fig. 3a-d) and have lateral pneumatic openings that are sometimes divided by a septum. The postzygodiapophyseal laminae of the middle and posterior cervicals extend far laterally along the posterior margins of the diapophyses, unlike most other sauropod taxa where these laminae fade out closer to the neural spine (Fig. 3a-d). All preserved cervicals possess pre-epipophyses^35^ and post-epipophyses as in taxa such as *Euhelopus*, *Jobaria*, *Turiasaurus* and *Moabosaurus* ^17^. Two character states in the cervical neural arch are regarded as local autapomorphies: first, a well-developed spinoprezygapophyseal lamina (SPRL) extends anteriorly onto the lateral surface of the prezygapophysis, and second, a lateral fossa lies below the SPRL. These character states are shared with some diplodocids (e.g. *Kaatedocus*)^26^. The cervical neural spines are single or have a very shallow bifurcation (Fig. 3k), differing from *Turiasaurus* which has more deeply bifid spines^1^. The cervical ribs are simple, differing from *Turiasaurus* and *Moabosaurus* ^17^ wherein the ribs have an accessory process projecting posterodorsally from the dorsal margin of the distal shafts^1^. The cervical ribs of *Mierasaurus* bear a bulge on the lateral surface of the tuberculum, immediately posterior to the base of the anterior process (a potential autapomorphy).

The dorsal centra of *Mierasaurus* lack complex lateral pneumatic openings and are amphicoelous in posterior elements of the series, differentiating *Mierasaurus* from *Moabosaurus* ^17^, *Camarasaurus* and titanosauriforms wherein these vertebrae are strongly opisthocoelous^3,27^. Middle and posterior dorsal neural spines have a posterior spinodiapophyseal lamina (post.SPDL) (Fig. 4h-l) that bifurcates dorsally such that a thinner posterior branch extends to the aliform process as usual, and a stouter anterior branch extends anterodorsally to the posterior surface of the anterior spinodiapophyseal lamina (ant.SPDL) (Fig. 4h-l): this additional anterior branch thus divides the spinodiapophyseal fossa (SDF) (Fig. 4h-l) into upper and lower portions, with the upper portion forming a dorsally opened fossa. The sacrum is only represented by sacral ribs formed from thin plates of bone with an expanded sacral articulation. Five different sacral rib morphotypes are present, suggesting that there were at least five vertebrae in the sacrum of *Mierasaurus*. The orientation of the dorsally-directed plate of bone on each sacral rib, and the posterior extension of the sacral articular area, demarcates a fossa on the ventromedial part of the dorsal plate, situated between the latter and the sacral articulation.

The anterior caudal vertebral centra are strongly procoelous, differing from the amphicoelous caudals of *Haplocanthosaurus*, *Camarasaurus* and many other sauropods, and are more similar to those of the non-neosauropods *Mamenchisaurus*, *Turiasaurus, Losillasaurus* ^1,3,27^, *Moabosaurus* ^17^ and most titanosaurs^3,27^. The caudal vertebral centra of *Mierasaurus* lack a ventral fossa and lateral pneumatic openings, differentiating them from those of diplodocids^26,29^. Caudal neural arches are situated on the anterior half of each centrum, (a derived state characterising most titanosauriforms^36^) with the neural spines directed posterodorsally. The anterior haemal arches are generally unbridged proximally, a derived state that occurs in *Shunosaurus*, *Haplocanthosaurus*, rebbachisaurids and macronarians^3,27^ although one element is nearly closed (Fig. 5g,h). The length of the haemal canal versus chevron total length is 41%, a derived condition shared with Titanosauriformes^34,37^.

The scapula has a rounded proximal plate with smooth acromial ridge and a long, fairly straight blade. The latter is slightly expanded distally (Fig. 5n). The sternal plate has a straight lateral margin and convex medial one, and is longer anteroposteriorly than transversely. The radius and ulna were preserved in articulation (Fig. 5j). These elements are straight and have transversely expanded distal ends. On the ulna, beside the fossa for the radius, there is a second deep proximolateral fossa on the lateral surface, similar to the derived condition seen in *Turiasaurus*, *Losillasaurus* (R.R.T. personal observation) and *Dystrophaeus* ^38^. The metacarpals are closely appressed to each other (Fig. 5m). Metacarpal I has two distal condyles, unlike *Giraffatitan* ^33^, and it is longer than metacarpal IV (as in many macronarians^27^).

The preacetabular process of the ilium has a subtriangular outline in lateral view (Fig. 5e), which is the plesiomorphic state that occurs in non-titanosauriforms^36^. As in neosauropods, this process projects anterolaterally^36^. The pubis lacks a prominent ambiens process. The ischia are short (Fig. 5d), with an ischium:pubis length ratio of 0.75, which has otherwise only been observed in titanosaurs^36^. The short ramus and small acetabular margin of the *Mierasaurus* ischium are also reminiscent of those of titanosaurs such as *Rapetosaurus* ^37^. The femur has a straight shaft (Fig. 5a) and its proximal head projects medially. The fourth trochanter is represented by a ridge and a notch on the posteromedial margin, located very proximally (i.e., the most ventral point of the fourth trochanter lies at 40% of femur length from the proximal end, whereas in other sauropods it is usually located just above midlength^3^). The distal femoral condyles are subequal in size (Fig. 5k). The absence of a lateral femoral bulge supports the placement of *Mierasaurus* outside Titanosauriformes^3,27,36^. The tibia has an anteroposteriorly elliptical proximal head and the cnemial crest projects anterolaterally. The posterior rim of the ascending process of the astragalus terminates a short way anterior to the posterior margin of its main body, which is the plesiomorphic state that occurs in non-neosauropod eusauropods^3,27^. The medial part of the astragalus forms a distinct subtriangular process in dorsal or ventral view, similar to the astragalus of *Turiasaurus*. This means that the medial one-third of the astragalus narrows in anteroposterior width such that it is about one-third of the width of the middle and lateral portion. The pedal phalangeal formula for *Mierasaurus* is 2-3-3-2-?0. Pedal unguals on digits II and III are unusual because they are compressed dorsoventrally; such a morphology has been observed in a few other sauropods, e.g. *Vulcanodon* ^39^ and possibly *Sanpasaurus* ^40^, but in most forms, these unguals are laterally compressed^41^.

### Phylogenetic results and comparison

Phylogenetic analysis (Fig. 6 and Supplementary Figs S4-S5) places both *Mierasaurus* and the previously established taxon *Moabosaurus* within the Turiasauria (a clade outside of Neosauropoda) and indicates that, these represent the only known non-neosauropods to have survived into the Cretaceous and the only turiasaurs known from North America. The results support the monophyly of Turiasauria that includes *Losillasaurus*, *Turiasaurus* and *Zby* from the Late Jurassic of the Iberian Peninsula^1,9,10^ and *Mierasaurus* and *Moabosaurus* from the Early Cretaceous of Utah, USA. The phylogenetic analyses identify several synapomorphies of Turiasauria in *Mierasaurus* (see Supplementary Information for a full list). This view is strengthened by other morphological features that have not yet been incorporated into phylogenetic data sets. For example, a deep proximolateral fossa in the ulna is present in *Turiasaurus*, *Losillasaurus*, *Zby*, *Moabosaurus* (R.R.T. personal observation in 2016) and *Mierasaurus*. *Turiasaurus* and *Mierasaurus* both possess a pair of small pits or foramina on the supraoccipital, one on either side of the dorsal end of the nuchal crest^9^, and the medial one-third of the astragalus narrows in anteroposterior width to form a distinct subtriangular process. However, *Mierasaurus* can be clearly distinguished from other turiasaurs via a suite of autapomorphies, as well as differences in other character states. For example, none of the cervical ribs in *Mierasaurus* are bifid, differing from *Moabosaurus* and *Turiasaurus*; the anterior cervical ribs of *Mierasaurus* have a small dorsal process projecting from the main distal shaft (i.e., BBGI-5D), potentially representing the incipient phase of development of bifid cervical ribs or the loss of this character, seen in some anterior ribs of *Turiasaurus riodevensis* paratype^1^ and in anterior and middle cervical ribs of *Moabosaurus* ^17^.

A comparison with other sauropods from the Early Cretaceous of the USA suggests that *Mierasaurus* is a distinct taxon. For example, *Mierasaurus* lacks the pneumatic openings in the proximal parts of the thoracic ribs that occur in *Astrodon* from the Arundel Formation of Maryland^42^. The anterior middle caudal neural spines of *Cedarosaurus* and *Venenosaurus* (Cedar Mountain Fm, Utah) are deflected anteriorly^43–45^ whereas in *Mierasaurus* they are deflected posteriorly. *Sauroposeidon* differs from *Mierasaurus* because the latter has shorter cervical vertebrae^46,47^. Compared to *Abydosaurus* and *Sonorasaurus*, *Mierasaurus* differs in the absence of camellate pneumaticity in its cervical and dorsal vertebrae^45,48^. *Brontomerus* has a longer and more rounded preacetabular process in the illium (a derived state characteristic of Titanosauriformes^47,49^), whereas *Mierasaurus* retains the plesiomorphic shorter and sub-triangular process. In addition, a detailed comparison of *Mierasaurus* and *Moabosaurus* ^17^, reveals numerous differences in the skull and postcranial skeleton: 1,) *Moabosaurus* lacks an occipital condyle with a pair of rounded ridges which is a diagnostic character for *Mierasaurus*; 2,) the tooth crowns in lingual view have vertical ridges in *Moabosaurus* that are not developed in *Mierasaurus*; 3,) the atlas of *Moabosaurus* has a robust parapophysis that is not present in *Mierasaurus*; 4,) the atlas intercentrum of *Moabosaurus* lacks the two posterolaterally facing depressions seen in *Mierasaurus*; 5,) *Mierasaurus* lacks bifid cervical ribs; 6,) *Mierasaurus* lacks the ventral hollows and keel on the ventral surface of the cervical centra that occur in *Moabosaurus* ^17^; 7,) the development of the CPRLs on anterior cervical vertebrae is different - convex in *Moabosaurus* and straight in *Mierasaurus*; 8,) The cervical ribs of *Moabosaurus* lack ridges or bulges on the lateral surface of the tuberculum, immediately posterior to the base of the anterior process; 9,) the PRSL is present in the middle and posterior dorsal vertebrae of *Moabosaurus* and absent in all dorsals of *Mierasaurus*; 10,) the morphology of the hyposphene differs in posterior dorsal vertebrae, rectangular in *Moabosaurus*, and triangular in *Mierasaurus*; 11,) all dorsal vertebrae of *Moabosaurus* are opisthocoelous whereas they are amphicoelous in the posterior dorsal vertebrae of *Mierasaurus*; and 12,) *Moabosaurus* has a slight lateral bulge on the proximolateral femur, which is completely absent in *Mierasaurus*. The other diagnostic characters of *Mierasaurus* can not be compared with *Moabosaurus* at present because of missing data, damage and/or lack of anatomical overlap.

These two North American Early Cretaceous turiasaurs share two synapomorphies that are not observed in their European relatives: 1,) a distinct, small, anteriorly projecting bump is present on the anterolateral part of each basal tuber, just above the distal tip, giving the tips of the tubera a fat L-shape in ventral view; and 2,) middle and posterior dorsal neural spines bear a posterior spinodiapophyseal lamina that bifurcates towards the summit, sending a posterior branch to the aliform process and an anterior branch to the anterodorsal margin of the spine. Therefore, *Mierasaurus* and *Moabosaurus* appear to be closely related but distinct, members of the Turiasauria.

## Discussion

There is some evidence that populations of sauropods undertook seasonal migrations of several hundred kilometers in order to locate appropriate environments and access food and water resources over a wide region^50^, and migrations allowed dinosaurs to explore new geographic areas^51^. In this way, it seems likely that sauropod groups were able to disperse widely provided that suitable land routes were available^52^.

The identification of *Mierasaurus* and *Moabosaurus* as turiasaurs has palaeobiogeographic implications. During the Late Jurassic, neosauropods were very abundant in North America: the Morrison Formation has yielded more than 430 specimens^53^. Virtually all of these belong to Diplodocoidea or Macronaria^3,27^. Even Late Jurassic North American taxa with uncertain affinities, such as *Haplocanthosaurus*, have been placed within Neosauropoda in most previous analyses^3,27,28^ and by the current study, so no confirmed non-neosauropod sauropods have ever been described in the Morrison Formation^3,27,53^. The development of a terrestrial dispersal route between Europe and North America after the late Tithonian could explain the absence of turiasaurs in the rich sauropod faunas of the Late Jurassic (Morrison Formation) of North America, and their presence during the Early Cretaceous (Yellow Cat Member of the Cedar Mountain Formation) in North America. The Jurassic/Early Cretaceous fossil record of eastern North America (Appalachia) has produced currently no evidence of turiasaurs: in fact, sauropod taxa from the Early Cretaceous of the eastern North America are very different, as explained above. Post -Tithonian-pre-Aptian sauropod faunas from North America have only been described from the Lakota Formation (Berriasian-Barremian) on the basis of a left humerus and the first metacarpal I (FMNH PR 487), both identified as Neosauropoda indet^54^. Thus, the pre-Aptian Cretaceous fossil record for sauropods is particularly poor^47,54^. This emphasises the value of the insights which *Mierasaurus* provides into Early Cretaceous North American sauropod faunas. Moreover, this poor fossil record makes it impossible to determine whether the sudden appearance of turiasaurs in Utah in the Early Cretaceous involved a dispersal from an Appalachian fauna during the Earliest Cretaceous or even Late Jurassic, though any such dispersal from Iberia is likely to have passed through this region. Future fieldwork must test the presence or absence of turiasaurs in eastern North America for the Late Jurassic and Early Cretaceous in terrestrial sediments.

During the early Mesozoic, North America and Europe were connected to form the western and central part of the supercontinent Laurasia. With the opening of the North Atlantic during the Late Jurassic and earliest Cretaceous, the region fragmented into smaller tectonic units^55–58^ that might have periodically formed contiguous land masses^57,58^ during sea level low-stands, characterized by exposure of continental margins and draining of continental interior seaways^55–58^. The presence of a land bridge between North America and Europe during the late Kimmeridgian-earliest Tithonian^13^ might explain similarities in some dinosaurs such as *Stegosaurus* ^59^ and *Allosaurus* ^60^. However, the absence of turiasaurs in the Morrison Formation suggests that this clade dispersed into western North America after the mid-Tithonian. Thus, unless we wish to argue that turiasaurs were actually present in the Morrison Formation but have not been found yet, a second later land connection between Europe and North America is required. Specifically, during the mid-Valanginian (*c*.137 Ma), a dramatic drop in sea level^7,55,56^ potentially reconnected Europe and North America and so permitted a final exchange of vertebrate clades across the Atlantic. This Valanginian event could also explain apparent faunal exchanges between these continents proposed for the late Hauterivian-early Barremian^13^. Taxa that seem to disperse in the Hauterivian/Barremian might actually have done so during the Valanginian lowstand, but gaps in the fossil record mean that the results of these dispersals might not be detected until we access better sampled post-Valanginian deposits. Aside from *Mierasaurus*, this connection is supported by the discovery of other groups in Utah, for example, a new haramiyidan mammaliaform (in a preliminary report in a conference abstract)^61^, a clade now documented in North Africa and Europe during the Jurassic–Cretaceous transition.

Thanks to recent discoveries, there has been a rapid growth in our knowledge of the turiasaur sauropods, both in terms of their evolution (e.g., the existence of a group of gigantic sauropods in Europe) and paleobiogeography (e.g., turiasaurs dispersing from Europe to North America in the Early Cretaceous). Many Early and Middle Jurassic sauropod faunas were dominated by various non-neosauropod eusauropod clades (broad-tooth-crown clades) and, in the Late Jurassic, the neosauropod diplodocoids and macronarians. At, or close to, the Jurassic-Cretaceous boundary, sauropods underwent a major extinction^4–7^. Cretaceous sauropod faunas were different from those of the Jurassic, the former being dominated by narrow-tooth-crowned clades (diplodocoids and titanosauriforms) from the late Early Cretaceous onwards^5,48^. In North America, the diplodocoids, so abundant and diverse in the Morrison Formation, disappeared at the Jurassic-Cretaceous boundary and the Cedar Mountain Formation is dominated by “brachiosaurid” titanosauriforms and camarasauromorphs that might have been descended from Morrison taxa. Turiasaurs, therefore, represent an interesting anomaly, demonstrating that a clade of broad-toothed non-neosauropods persisted beyond the Jurassic-Cretaceous extinction and found at least temporary refuge in North America.

## Methods

### Fieldwork

The Utah Geological Survey has been excavating the Doelling’s Bowl bonebed (DBBB) in east-central Utah annually since 2005 (Supplementary Figs S1-S3). The DBBB is a multitaxic bonebed that crops out in an area of low relief and is estimated to cover an area of over 5000 m^2^. Over 1500 individual vertebrate bones have been excavated, mapped (Fig. 1), and collected to date, from several excavation areas totaling 140 m^2^. The bones are found in a green-gray sandy mudstone with traces of silcrete and sparse root casts along with abundant chert pebbles. In 2010, a partially articulated sauropod dinosaur was discovered eroding out of an arroyo in an area of the bonebed dubbed Gary’s Island. The articulated manus and pes extended through the sediment at an angle below the level of the majority of the skeleton, indicating that an individual sauropod became mired in soft sediment and died in place. After decomposition, many of the elements of the skeleton were scattered over an area of roughly 10 m^2^. Several shed theropod teeth have been found in association with the sauropod skeleton but no evidence of tooth marks has yet been identified on any of the bones from this part of the quarry. There is minor plastic deformation of many of the bones that, along with sedimentological evidence, suggest that the area was waterlogged. The undersides of the recovered bones show more degradation than the upper surfaces, possibly as a result of grazing by invertebrates. The material has been cleaned and prepared in the laboratory of the Utah Geological Survey. Exact locality information will be provided to qualified researchers on request through the Natural History Museum of Utah or the Utah Geological Survey.

### Phylogenetic analysis

We conducted phylogenetic analyses using two different data matrices^62,63^ utilizing TNT 1.1^64^ in order to find the most parsimonious trees (MPTs). For the pruned Carballido and Sander matrix^62^, we implemented a heuristic tree search in TNT. This performed 1000 replications of Wagner trees (using random addition sequences) followed by tree bisection reconnection as the swapping algorithm, saving 10 trees per replicate (Supplementary Fig. S4). For the Mannion *et al*. analysis^63^, the pruned data matrix was analysed using the Stabilize Consensus option in the New Technology Search in TNT 1.1^64^. Searches were carried out using sectorial searches, drift, and tree fusing, with the consensus stabilized five times, prior to using the resultant trees as the starting trees for a traditional Search using Tree Bisection-Reconnection (Supplementary Fig. S5). In order to test the support of the phylogenies, the Bremer support and the bootstrap values were obtained (absolute frequencies based on 10,000 replicates).

## Electronic supplementary material

Supplementary Information
