# Supplementary material for: Descendants of the Jurassic turiasaurs from Iberia found refuge in the Early Cretaceous of western USA
Source: Sci Rep. 2017 Oct 30;7:14311. doi: 10.1038/s41598-017-14677-2 (PMC5662694; doi:10.1038/s41598-017-14677-2)
Supplement: Supplementary file 1 — Supplementary Information [file 41598_2017_14677_MOESM1_ESM.pdf]

Descendants of the Jurassic turiasaurs from Iberia found refuge in the Early Cretaceous of western USA

Rafael Royo-Torres, Paul Upchurch, James I. Kirkland, Donald D. DeBlieux, John R. Foster, Alberto Cobos, Luis Alcalá

Supplementary Information:

I. Abbreviations section

II. Geological setting of the Doelling's Bowl bonebed

III. Phylogenetic analyses and list of synapomorphies

IV. Holotype of *Mierasaurus*

V. Measurements

VI. References

I. Abbreviations section

BYU (Museum of Paleontology of Brigham Young University, Provo, Utah USA). DBBB Doelling's Bowl bonebed; DBGI Doelling's Bowl site; J-K Jurassic-Cretaceous transition; Ma million years ago; My million years; MPT most parsimonious trees; RD name for every dinosaur site from Riodeva in Teruel (Spain); TL Tree length; UMNH the Natural History Museum of Utah (USA); UGS Utah Geological Survey (USA). Anatomical abbreviations: bt basal tubera; bo basioccipital; bpt basipterygoid process; ca crista antotica; ct crista tuberalis; f frontal; fme fenestra metotic; fv fenestra vestivuli (=fenestra ovalis); osc otosphenoidal crest (= crista prootica); p parietal; pa parapophysis; prf prefontal; stf supratemporal fossa; so supraoccipital; eo exoccipital-opisthotic; bo basioccipital; popr paraoccipital process; cranial nerves: I olfactory nerve; II optic nerve; III oculomotor nerve; IV trochlear nerve; V trigeminal nerve (ophthalmic, maxillary, mandibular); VI abductor or abducens nerve; VII facialis nerve; VIII vestibulocochlear nerve. Abbreviations for laminae vertebral: acpl anterior centroparapophyseal lamina; ant. spdl anterior spinodiapophyseal lamina; cprl

centroprezygapophyseal lamina; cpol centropostzygapophyseal lamina; pcdl posterior centrodiapophyseal lamina; post. spdl posterior spinodiapophyseal lamina; ppdl paradiapophyseal lamina; prdl prezygodiapophyseal lamina; spol spinopostzygapophyseal lamina.

II. Geological setting of the Doelling's Bowl bonebed (UMNH Loc. 1208)

The Lower Cretaceous Cedar Mountain Formation of east central Utah spans 30-35 My and has been subdivided into six members (Fig. S1) based on lithostratigraphic differences, sequence stratigraphy supported by vertebrate biostratigraphy, and supplemented by radiometric dates. In ascending order, these are the Buckhorn Conglomerate Member, Yellow Cat Member, Poison Strip Member, Ruby Ranch Member, Short Canyon Member, and Mussentuchit Member[1-4]. The Yellow Cat Member at the base of the Cedar Mountain Formation is restricted to Grand County in eastern Utah as a result of local Early Cretaceous subsidence due to salt tectonics in the northern Paradox Basin[3,5]. The Yellow Cat Member is informally divided into lower and upper units separated by a regionally widespread calcrete formerly used to mark the boundary of the Upper Jurassic Morrison Formation with the overlying Lower Cretaceous Cedar Mountain Formation[2,6,7]. The discovery of a distinctive dinosaur fauna of Lower Cretaceous aspect including the troodont *Geminiraptor*[8], the therizinosaur *Falcarius*[9-11], the dromaeosaurid *Yurgovuchia*[12], the basal styracosternan iguanodont *Iguanocolossus*[13], undescribed species of polacanthid ankylosaurs[3], and indeterminate sauropods[3] resulted in a redefinition of the boundary to include the stacked pebble-bearing, ferruginous paleosols preserving these new Cretaceous dinosaurs[3,5,14,15]. Overlying the calcrete, the upper Yellow Cat Member is characterized by the dromaeosaur *Utahraptor*[16], the iguanodonts *Hippodraco*[13] and an undescribed sail-backed taxon[2], the polacanthid ankylosaur *Gastonia*[17] and the brachiosaurid titanosauriform sauropod *Cedarosaurus*[18]. *Mierasaurus* is only known from the lower Yellow Cat Member of the Cedar Mountain Formation in the Doelling's bowl bonebed (UMNH Loc. 1208). The Doelling's bowl bonebed can be precisely correlated to the type Yellow Cat section a few kilometers to the west (Fig. S2).

The length of time represented by the hiatus between the Morrison and Cedar Mountain Formations is impossible to determine accurately at this time. The youngest ages for Morrison strata in this area range from 148.96 +2.54 -2.21 Ma [19] based on U-Pb ages determined by laser ablation of zircons extracted from a volcanic ash bed about 8.7 meters below the upper contact of the formation to a recalibrated $^{40}Ar/^{39}Ar$ age 150.00±0.52 Ma from near the top of the Brushy Basin Member[20]. Thus, the top of the underlying Morrison Formation dates to the lower third of the Tithonian Stage near to, but not to the end of, the Jurassic[21,22].

Ages from the Yellow Cat Member of the Cedar Mountain Formation are limited to U-Pb maximum ages determined by laser ablation of detrital zircons. The most widely cited maximum age of 124.2 ± 2.6 Ma[23-25] is from a sandy green mudstone near the base of the Yellow Cat Member. Older ages for the lower Cedar Mountain Formation have recently been proposed based on ostracodes and charophytes extracted from the upper part of the Yellow Cat. These ages are based largely on the comparison of these microfossils with those known from the Berriasian Purbeck Formation of southern England[26-28]. Furthermore, U-Pb maximum ages from detrital zircons collected from paleosols in which a minimum of 300 zircon grains were dated have suggested maximum dates ranging from 137 to 139 Ma from throughout a section of the Yellow Cat Member at *Utahraptor* Ridge north of Arches National Park [3,29]. A maximum age of 139.7 ± 2.2 Ma age (based on two youngest zircon grains of 300 dated) occurs in the lowest paleosol in the section and is the oldest non-Jurassic maximum age so far extracted from the basal Cedar Mountain Formation. Another detrital zircon age from the Doelling's bowl bonebed in the lower Yellow Cat Member yielded a maximum age of ~132 Ma[3].

The disparities of ages for the Yellow Cat Member require further investigation. What they do suggest is that the time missing across the K-1 unconformity in the northern Paradox Basin of east-central Utah ranges from a maximum of 26 Ma to as little as 10 Ma. Numerous periods of pedogenesis in the lower Yellow Cat Member are likely to have accumulated a range of zircon populations and "pedoturbation" may have caused mixing of these zircons[3]. Preliminary unpublished paleomagnetic data indicate that deposition of the Yellow Cat Member, at least in part, preceded the onset of the Cretaceous Long-Normal at the base of the Aptian[30,31].

The occurrence of different dinosaur taxa in the upper and lower Yellow Cat raises the possibility of testing the hypothesis that the calcrete, although not representing the K-1 unconformity, at a minimum, represents a hiatus spanning evolutionary time as dinosaur genera turned over fairly rapidly, on the order of every 1 to 10 million years[32-36]. The discovery of *Mierasaurus bobyoungi* provides important supportive data in the separation of the upper and lower Yellow Cat dinosaur faunas. Researchers at Brigham Young University are studying abundant sauropod material from more than a dozen individuals of a closely related sauropod, *Moabosaurus utahensis*[37] found with *Gastonia* and *Utahraptor* in the Yellow Cat Member at the Dalton Wells Quarry north of Moab, Utah[24]. Differences in character states between these sauropods provide an additional test of our hypothesis. Probably the most noticeable difference in character states is that the middle cervical ribs deepen distally in *Mierasaurus* and are forked distally in *Moabosaurus*[37]. The dinosaurs, together with pollen and charophytes (green algae), and various isotopic dating methodologies suggest that the Yellow Cat Member rocks are anywhere from 142 to 124 million years old[2,3,7,24,25,29].

The Doelling's bowl bonebed (UMNH Loc. 1208) covers an area of approximately 90,000 m^2, although the area of shallowly buried disarticulated and associated dinosaur skeletons on the east side is only about 1000 m^2 (Fig. S3, S4). Excavations have only been conducted a few weeks per year over the past 10 years, but have resulted in the removal of several thousand bones representing dozens of individuals[38]. In addition to *Mierasaurus*, the Doelling's bowl bonebed preserves the type specimen of the dromaeosaurid *Yurgovuchia*, large shed teeth of an allosauroid theropod, abundant juvenile to sub adult skeletons of the basal steracosternid iguanodont *Iguanocolossus*, and several examples of an undescribed polacanthid ankylosaur. Non-dinosaurian remains include skull fragments and teeth that may pertain to aquatic goniopholidid crocodyliforms and distinctive shell fragments of a turtle similar to *Naomichelys*[3].

Skeletal remains occur at several levels within the approximately one meter thick bonebed interval. The bonebed is in the medial part of the lower Yellow Cat Member 9.5 to 10.5 m above the K-1 unconformity. The bonebed directly overlies a laterally extensive interval of thin (1 cm) to locally thick (0.4-1.0 m thick) chert beds with

silicified fern roots that might represent silicified peat mats and silcrete spring deposits derived from silica-saturated water flowing up from the underlying Morrison Formation. Similar beds occur near the top of the Sonsela Member ("persistent red silcrete zone") of the Upper Triassic Chinle Formation at Petrified Forest National Park[39], where they are associated with faunal and floral turnover at the Late Triassic (Norian) Adamanian–Revueltian faunal and floral transition[40]. This Chinle silcrete zone is interpreted to represent the silica replacement of peat (histosols) and pedogenetic carbonate in an interval recording a pronounced shift from humid to overall drier climates as indicated by abundant pedogenetic carbonate nodules up section[41]. The early formation of these cherts is indicated by the presence of rounded silicified fern root fragments among the dinosaur bones at Doelling's bowl.

The bone-bearing strata consist of mudstone with common floating chert pebbles derived from the underlying Morrison Formation, silicified root fragments and rare rounded dinosaur bone fragments. These rocks appear relatively flat bedded, but in actuality form broad swales over tens of meters. The most common skeletal remains are those of juvenile, basal styracosternan iguanodonts, followed by the new polacanthid ankylosaur and dromaeosaurid remains. Discovered in 2010, *Mierasaurus* was restricted to a small area on the western end of the excavated portion of the bone bed. Most of the bones pertain to a single individual that was trapped in mud as indicated by the articulated lower limbs extending below the level of the majority of the skeleton. The terminal 10 caudal vertebrae were also found in articulation. A few bones representing at least one additional individual are present; as indicated by the presence of three femora. Distinctively, the upper surfaces of the bones were better preserved than the lower surface, perhaps due to grazing invertebrates. This, together with horizontal root traces suggests a wet, boggy environment. A few isolated juvenile iguanodont and theropod bones were found scattered among the sauropod bones; as was the medial portion of a large articulated iguanodont tail. Approximately 25 cm below the mired feet of the sauropod, a lower bone-bearing surface contained a well-preserved associated skeleton of a large *Iguanocolossus* among scattered bones of other smaller individuals.

The *Mierasaurus* discovery was on the west side of a dry wash that likely removed a portion of the skeleton. As the beds preserving the majority of the bones

dipped east, the bone-bearing interval intersected the erosional surface 7-8 m to the west of the wash. Fortuitously, the majority of the skeleton was recovered, making the holotype of *Mierasaurus bobyoungi* the most complete single sauropod skeleton yet recovered from the Cretaceous of North America.

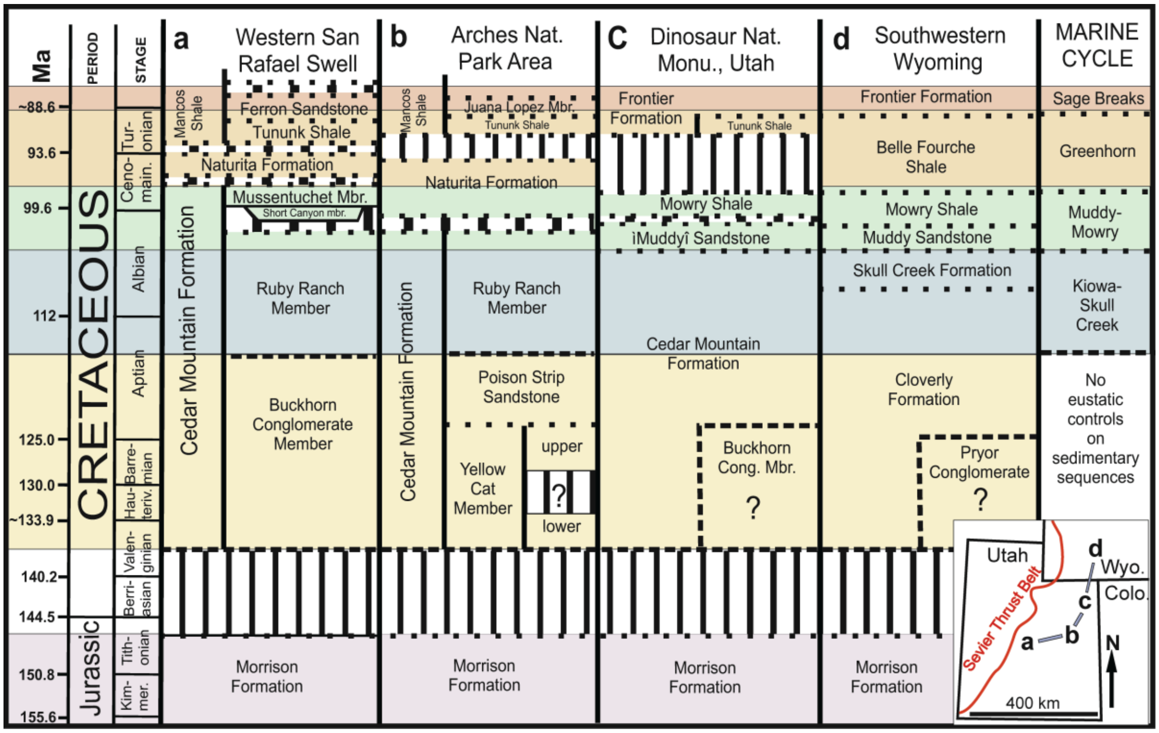

Fig. S1 The medial Mesozoic strata in eastern Utah with their correlation to contiguous strata in southwestern Wyoming showing the sequence stratigraphic relationships within these units. Naturita Formation is used for the strata overlying the Cedar Mountain Formation instead of the Dakota Formation following Young[3,41,42]. Figure created by J.I.K. (© Utah Geological Survey) in Adobe Illustrator CS5 (www.adobe.com/es/products/illustrator.html).

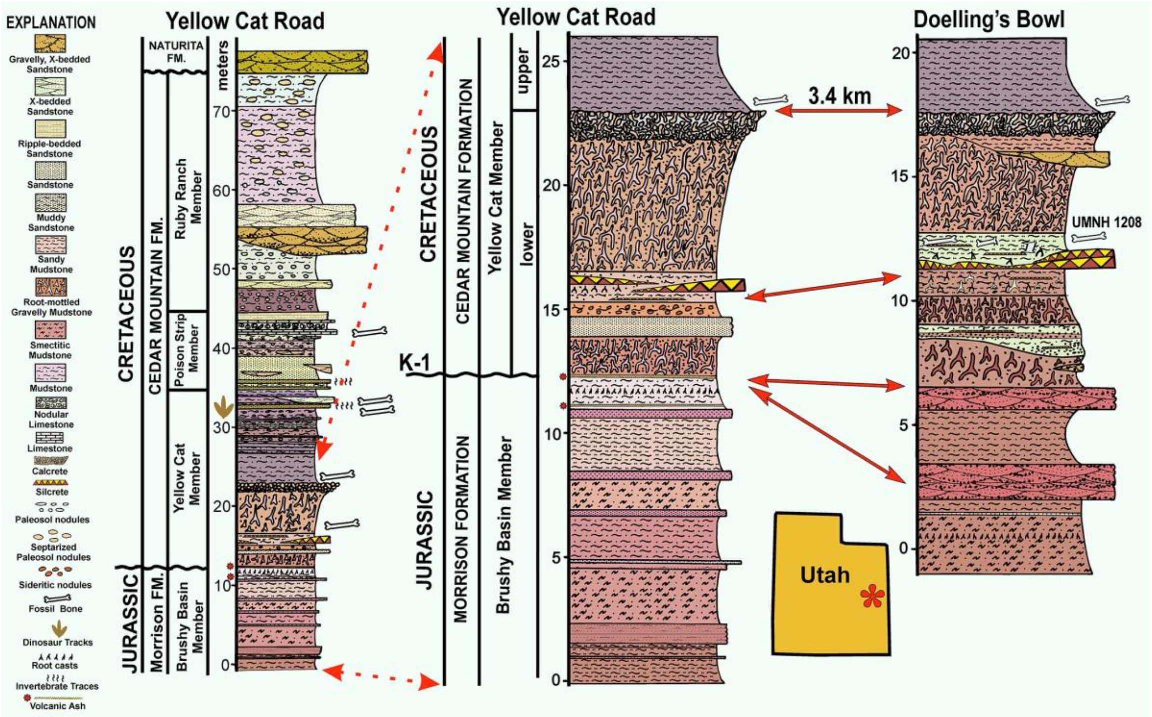

Fig. S2 The revised type section of the Yellow Cat Member of the Cedar Mountain Formation showing the correlation of the Doelling's Bowl bone (UMNH Loc. 1208) [12]. K-1 = Jurassic – Cretaceous boundary. Figure created by J.I.K and D.D.D. (© Utah Geological Survey) in Adobe Illustrator CS5 (www.adobe.com/es/products/illustrator.html).

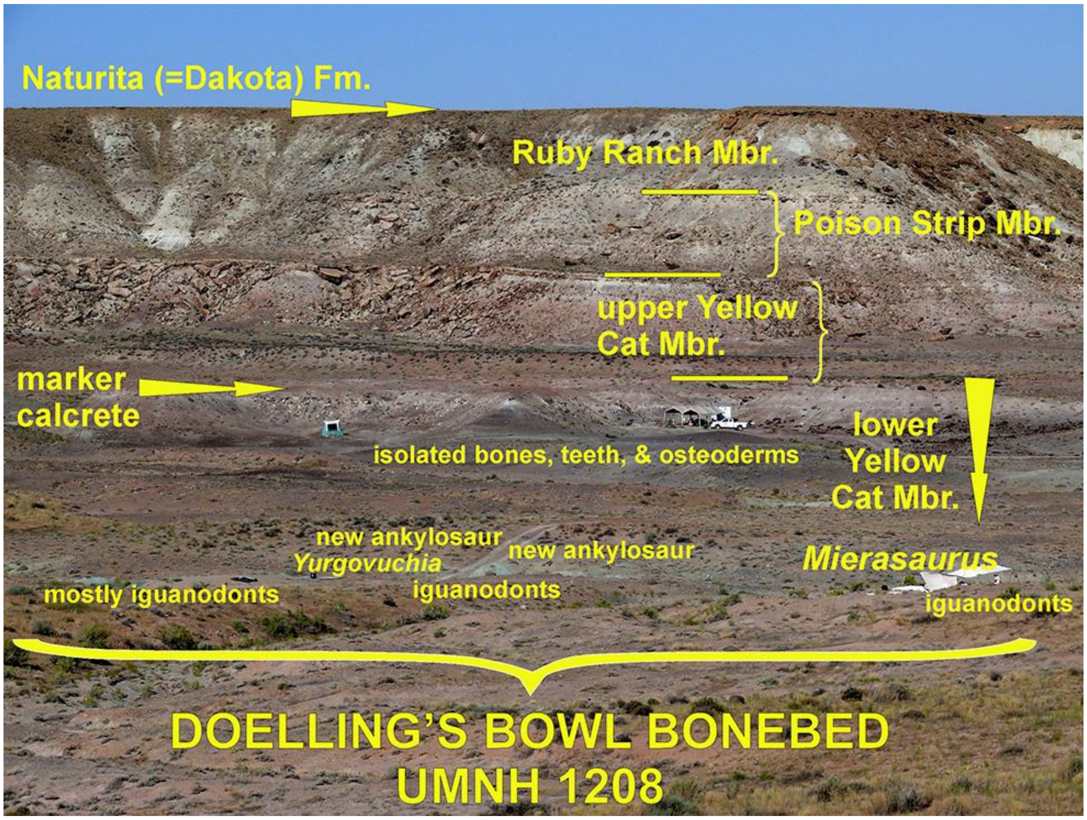

Fig. S3 Overview of Doelling's Bowl from the east showing the distribution of Lower Cretaceous units and the distribution of important fossil remains such as where *Mierasaurus bobyoungi* gen. nov., sp. nov. was excavated at Gary's Island (Utah, USA) by J.I.K and D.D.D. (© Utah Geological Survey) in Adobe Illustrator CS5 (www.adobe.com/es/products/illustrator.html).

III. Phylogenetic analyses

Carballido and Sander matrix[45]

Phylogenetic relationships of *Mierasaurus* gen. nov. were assessed on the basis of an analysis using an updated version of the Carballido and Sander matrix[45]. This analysis includes 73 taxa and 341 characters. Characters 12, 58, 95, 96, 102, 106, 108, 115, 116, 119, 120; 145, 152, 163, 213, 216, 232, 233, 234, 235, 252, 256, 298, 299 and 301 were treated as ordered multistate characters.

The following characters have been modified for *Turiasaurus* from those used by Carballido and Sander[45]: C40, ? changed to 1; C41, ? changed to 0; C43, ? changed to 0; C44, ? changed to 0; C45, ? changed to 0; C46, ? changed to 1; C47, ? changed to 0; C49, ? changed to 0; C50, ? changed to 0; C52, ? changed to 1; C53, ? changed to 1; C54, ? changed to 0; C55, ? changed to 0; C56, ? changed to 0; C57, ? changed to 1; C59, ? changed to 1; C65, ? changed to 0; C66, ? changed to 0; C67, ? changed to 0; C68, ? changed to 0; C69, ? changed to 0; C70, ? changed to 0; C71, ? changed to 0; C72, ? changed to 0; C73, ? changed to 1; C74, ? changed to 0; C75, ? changed to 0; C76, ? changed to 0; C78, ? changed to 0; C80, ? changed to 1; C87, ? changed to 1; C88, ? changed to 0; C89, ? changed to 1; C94, ? changed to 1; C95, ? changed to 1; C97, ? changed to 0; C98, ? changed to 1; C99, ? changed to 0; C100, ? changed to 1; C106, 0 changed to 2; C108, 1 changed to 0; C128, ? changed to 0; C130, 1 changed to 0; C133, ? changed to 0; C148, ? changed to 1; C149, ? changed to 0; C168, 1 changed to 0; C169, 1 changed to 0; C171, 0 changed to ?; C181, ? changed to 2; C182, ? changed to 1; C183, ? changed to 1; C184, ? changed to 0; C186, ? changed to 0.

The following characters have been modified for *Losillasaurus* from those used by Carballido and Sander[45]: C68, 1 changed to 0; C70, ? changed to 0; C71, ? changed to 0; C72, ? changed to 0; C73, ? changed to 1; C74, ? changed to 0; C95, 1 changed to ?; C98, 1 changed to ?; C280, 0 changed to ?.

The following characters have been modified for *Moabosaurus* from those used by Britt *et al.*[37] (P.U. and R.R.T. personal observation in BYU): C66, 1 changed to 0; C67, 1 changed to 0; C70, 1 changed to 0; C73, 0 changed to 1; C118, 1 changed to 0; C129, 1 changed to 0; C140, 0 changed to 1; C171, 0 changed to 1; C173, 1 changed to 0.

The data matrix was analyzed using TNT 1.1[46] in order to find the most parsimonious trees (MPTs). The result of the parsimony analysis yielded 1704 TL and 18 MPTs. The strict consensus closely matches the main topology obtained by Carballido and Sander[45] with Zby, *Mierasaurus* and *Moabosaurus* placed in the Turiasauria, outside of Neosauropoda.

Scoring of *Mierasaurus bobyoungi* for the characters of the modified Carballido and Sander matrix[45]

?????????????????????????????????0?????10?0?010??0?11000????????0000000010????
110000?1??????111010110110200?11111{02}10001000110?00000?100111111??10100
0000100011001111100010011001OO???????0????2010100100??001012102?01100?????
{01}(01)??1101000000001???????????????????11?01????110100111?11000101000010
01??0111000000010001?101010101?1110111111111101010

Scoring of *Zby*[47] for the characters of the modified Carballido and Sander matrix[45]

???
?????????????????0110110200??
??00??11??0?0?00?
0?10?00??????101001001110110??110000?01??????????????????????????????????????
?????????????????????????

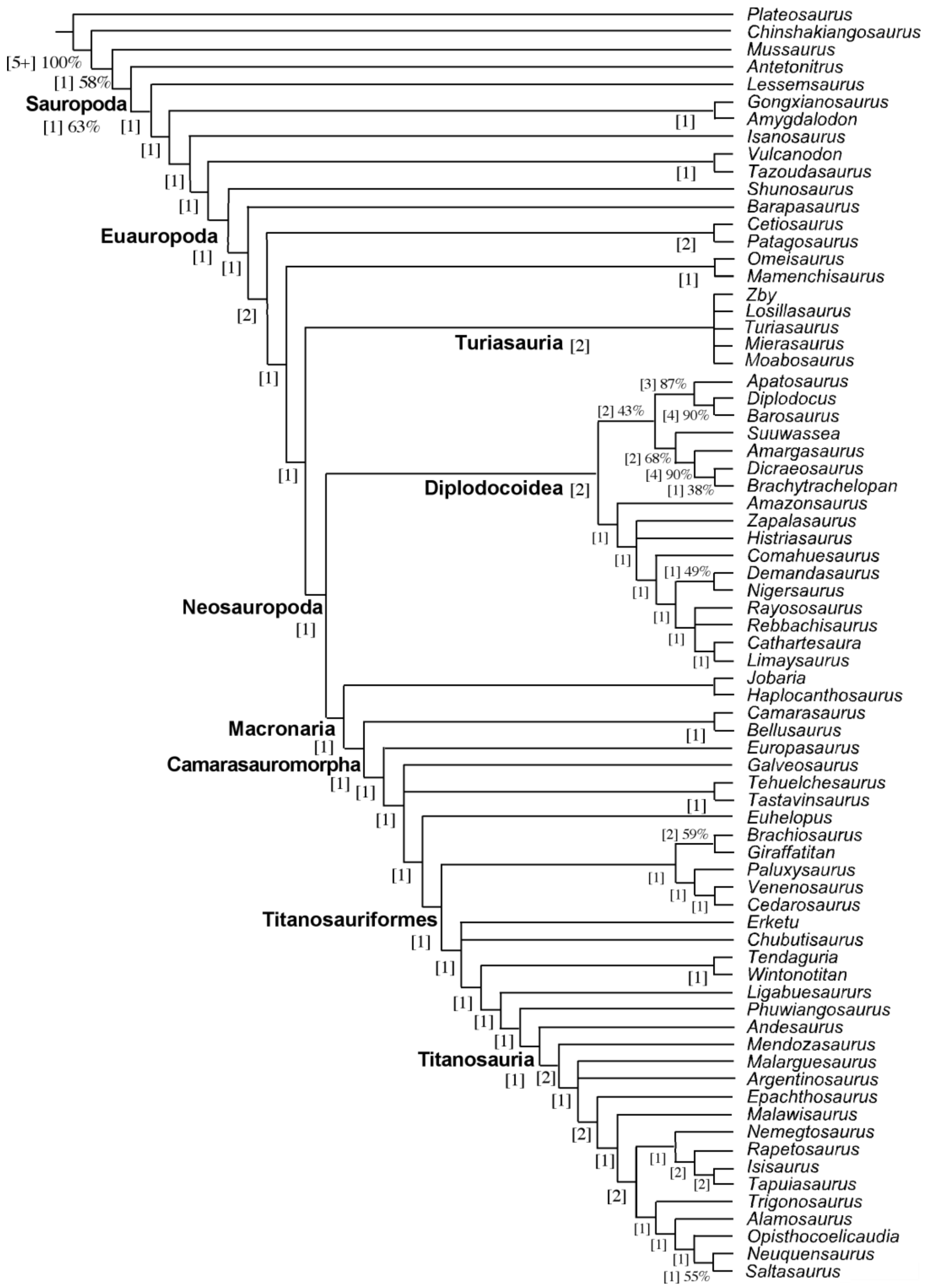

Fig. S4. Phylogenetic relationships of *Mierasaurus* gen. nov. based on an analysis using an updated version of the Carballido and Sander matrix[45]. Bootstrap values are shown as percentages (nodes lacking percentages have bootstrap values of less than 50%). Bremer supports are shown in square brackets.

Mannion *et al.* matrix[48]

Phylogenetic relationships of *Mierasaurus* gen. nov. and *Moabosaurus* were assessed on the basis of an analysis using an updated version of the Mannion *et al*. matrix[48]. This analysis includes 79 taxa and 415 characters. Character 413 was deleted because it is based on an erroneously described synapomorphie for Turiasauria[49: i.e.] the ulna of *Losillasaurus* and *Turiasaurus* have the vertical groove and ridge on the anteromedial surface of distal shaft as in the other sauropods (R.R.T. personal observation). Characters 11, 14, 15, 27, 40, 51, 104, 122, 147, 148, 177, 195, 205 and 259 were treated as ordered multistate characters, and nine unstable and highly incomplete taxa (*Astrophocaudia*, *Australodocus*, *Brontomerus*, *Fukuititan*, *Fusuisaurus*, *Liubangosaurus*, *Malarguesaurus*, *Mongolosaurus* and *Tendaguria*) were excluded a priori as in the original Mannion *et al*. analysis.

The following characters were modified for *Atlasaurus*[50]: C105, ? changed to 0; C108, 1 changed to 0; C110, ? changed to 0.

The following characters have been modified for *Losillasaurus* : C18, ? changed to 0; C19, ? changed to 1; C49, ? changed to 0; C132, ? changed to 0; C133, ? changed to 0; C141, 1 changed to 0, C150, ? changed to 0; C, 281, ? changed to 1.

The following characters have been modified for *Turiasaurus*: C18, ? changed to 0; C19, ? changed to 1; C20, ? changed to 1; C21, ? changed to 0; C24, ? changed to 0; C25, ? changed to 1; C27, ? changed to 2; C44, 1 changed to 0; C72, 0 changed to 1; C79, ? changed to 1; C104, ? changed to 0; C134, ? changed to 0; C137, ? changed to 0; C141, 1 changed to 0; C172, ? changed to 0; C174, ? changed to 0; C176, ? changed to 0; C178, ? changed to 0; C180, ? changed to 0; C192, ? changed to 1; C193, 1 changed to ?; C, 281, ? changed to 1.

The pruned data matrix was analysed using TNT 1.1, following the same protocol as in Mannion *et al.*[48]. The 'Stabilize Consensus' option in the 'New Technology Search' in TNT 1.1[46] was applied first. Searches were carried out using sectorial searches, drift, and tree fusing, with the consensus stabilized five times, prior to using the resultant MPTs as the starting trees for a 'traditional Search' using Tree Bisection-Reconnection.

This resulted in 24 MPTs of 1074 steps. The strict consensus closely matches the main topology obtained by Mannion *et al.*[48], with Zby, *Mierasaurus* and *Moabosaurus* placed in the Turiasauria, outside of Neosauropoda.

Scoring of *Moabosaurus urahensis* for the characters of the modified Mannion *et al*. matrix[48]

??0??0001?0???00001?01101?210101(01)0??????10???????00???????????????????????
????????????????10???0010?????0??000011?1110011100011100011000001010110021001000100000011001 0??????0?020?011110000?0?10?010001???0???00????????????000
000?0???0100??010?????????????????????
????????????????0??010001?0001100010100?????0101

Scoring of *Mierasaurus bobyoungi* for the characters of the modified Mannion *et al*. matrix[48]

000??0001?0???00001?00100121010???100???????1000?0?1?011??11000??0?????111?
0??100?0??0????10???0010???0000?000011?11000??1000111000000000010001100110
110001000000110000???00???020?011?10000???10??????????0???(01)0??000001?????
????????00??????0??0?000000000100000011000100???1?0?0?10001?0???????????????
?????????01?????????000?000001?0001000010100?????0?01????0?00?????????????01
1????1???00?0(0 1)0010??0??1???10000?????0???

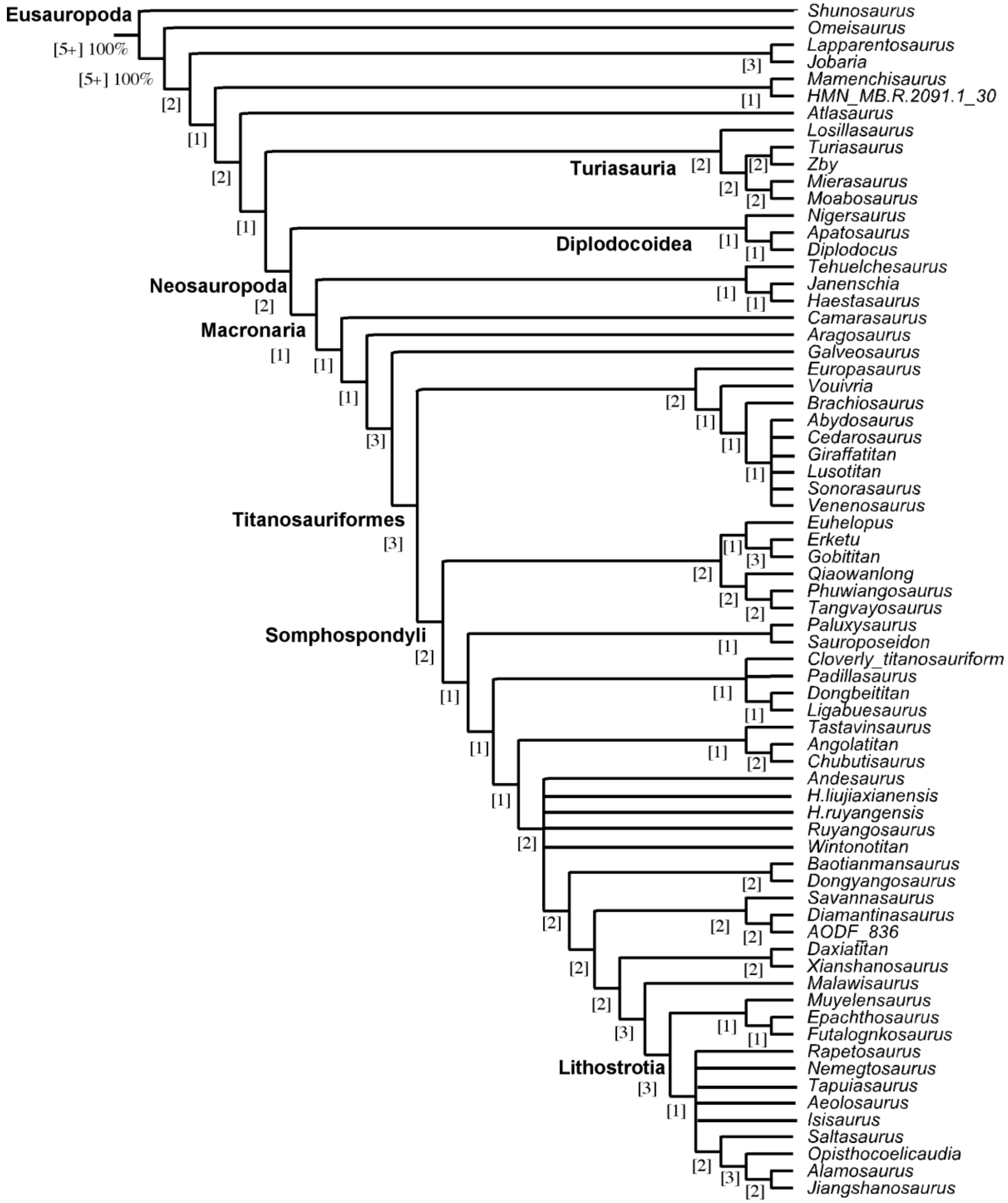

Fig. S5. Phylogenetic relationships of *Mierasaurus* gen. nov. based on an analysis using an updated version of the Mannion *et al*. matrix[48]. Bootstrap values are shown as percentages (nodes lacking percentages have bootstrap values of less than 50%). Bremer supports are shown in square brackets.

List of synapomorphies

Synapomorphies of Turiasauria, based on Carballido and Sander analyses[45]

Character (73). Basal tubera, shape of posterior face: slightly concave (1)

Character (171). Middle and posterior dorsal vertebrae, height of neural arch below the postzygapophyses (pedicel): subequal to or greater than height of centrum (1)

Character (174). Posterior dorsal centra, articular face shape: slightly opisthocoelous (1)

Character (193). Anterior caudal centra (excluding the first), articular face shape: slightly procoelous (2).

Character (197). Anterior caudal centra, pleurocoels: present (1)

Character (242). Coracoid, proximodistal length: approximately twice the length of scapular articulation (1)

Character (256). Humerus, RI: gracile (less than 0.27) (0)

Character (310). Tibia cnemial crest, orientation: projecting anteriorly (0)

Synapomorphies of Turiasauria, based on Mannion *et al.* analyses[48]

Character 28. Middle caudal centra, mediolateral width to dorsoventral height (excluding chevron facets) of anterior surface ratio: 1.0 or greater (1)

Character 32. Anteriormost caudal neural spines, dorsoventral height divided by centrum height: less than 1.2 (1).

Character 41. Humerus, maximum mediolateral width of proximal end divided by proximodistal length: less than 0.4 (1).

Character 281. Ulna, posterior process of proximal end: strongly developed, so that the proximal profile of the ulna is ‘T’- or ‘Y’-shaped, and there is a deep fossa between the anteromedial and posterior processes, rivalling the radial fossa in depth (1).

Character 402. Teeth, D-shaped crown morphology in labial/lingual view: narrows mesiodistally along its apical half, giving it a ‘heart’-shaped outline (1).

Character 412. Radius, proximal to distal end anteroposterior length ratio: 0.5 or greater (0).

IV. List of the holotype elements of *Mierasaurus*

Table S1. List of holotype specimens of *Mierasaurus bobyoungi* (UMNH.VP.26004) as keyed to field numbers (DBGI #).

**Element**	**Field number**
Braincase	DBGI 173
Premaxillla	DBGI 107D
Maxilla (two fragments)	DBGI 78A, 95I
Lacrimal	DBGI 160
Nasal	DBGI-85A
Right quadrate	DBGI 54
Jugal	DBGI 78B
Postorbital?	DBGI-30
Prearticular	DBGI 70
Surangular	DBGI 71
Left dentary	DBGI 60
Right dentary	DBGI 66
Premaxillary-maxillary tooth	DBGI 95
Posterior dentary tooth	DBGI 27
Anterior dentary tooth	DBGI 46
Atlas	DBGI 5I
8 cervical vertebrae	DBGI 19A, DBGI 38, DBGI 69G1, DBGI 69G2, DBGI 69H, DBGI 95, DBGI 165, DBGI 248
11 cervical ribs	DBGI 5D, DBGI 9, DBGI 10, DBGI 28A, DBGI 28B, DBGI 45F, DBGI 95A, DBGI 95B, DBGI 95C, DBGI 95D, DBGI 95H
11 dorsal vertebrae	DBGI 54A, DBGI 11, DBGI 16, DBGI 19A, DBGI 24B, DBGI 37, DBGI 100NA1, DBGI 100NA2, DBGI 115, DBGI 181A, DBHI 19B
6 dorsal ribs	DBGI 100 R1, DBGI 100R2, DBGI 100 R3, DBGI 100 R4, DBGI 100R5, DBSP 01
6 sacral ribs	DBGI 54B, DBGI 69B, DBGI 69I, DBGI 100SR1, DBGI 100SR2, DBGI 100SR3
15 caudal vertebrae	DBGI 5B, DBGI 5C, DBGI 23B, DBGI 34, DBGI 37B, DBGI 42A-E, DBGI 48, DBGI

	52A, DBGI 80, DBGI 100V2, DBGI 164, DBGI 192,
2 chevrons	DBGI 172, DBGI 252
Scapulae	DBGI 250, DBGI 260
Sternal plates	DBGI 1, DBGI 3
Left ulna and radius	DBGI 100R, U
Left complete manus	DBGI 100M
Left ilium	DBGI 100IL
Left pubis	DBGI 5A
Right pubis	DBGI 195
Right ischium	DBGI 69A
Left and right femur	DBGI 39, DBGI 100
Left tibia, fibula, astragalus and complete pes	DBGI 75

V. Measurements

Table S2. Holotype specimen of *Mierasaurus bobyoungi* from Doelling's Bowl bonebed (Utah, USA), measurements in mm.

	**Left femur**	**Left tibia**	**Left fibula**	**Left Mt I**	**Left Mt II**	**Left Mt III**	**Left Mt IV**	**Left Mt V**	**Left ungueal (manus) I**	**Left phalange (manus) II**	**Left phalange (manus) III**
Maximum length	1030	630	700	110	130	160	160	95	155	110	60

	**Right ischium**	**Right pubis**	**Right ilium**	**Right femur**	**Right scapula**	**Left ulna**	**Left radius**	**Left Mc I**	**Left Mc II**	**Left Mc III**	**Left Mc IV**
Maximum length	540	720	530	114	960	450	510	200	190	170	160

	**Left Mc V**	**Left phalange ungueal (pes)**	**Cervical ribs**	**Dorsal ribs**	**Chevron DBGI-172**
Maximum length	160	95	Between 240 and 500	Between 1200 and 720	235 (The notch 110)

	**Cervical vertebrae**	**Dorsal vertebrae**	**Anterior caudal vertebrae**	**Posterior caudal vertebrae**
Antero-posterior length	Between 170 and 250	Between 100 and 140	Between 6 and 50	Between 35 and 75

**Cervical vertebrae**	**High of the centrum**	**Width of the centrum**	**Length of the centrum**	**High of the neural arch**	**Total high**
**DBGI-69H**	70	-	215	100	170
**DBGI-69G1**	-	-	170	-	-
**DBGI-69G2**	60	90	200	120	180
**DBGI-165**	-	-	-	100	-
**DBGI-38**	90	110	200	90	180
**DBGI-19A**	190	70	250	-	240
**DGBI-95**	130	65	275	30?	-

**Dorsal vertebrae**	**High of the centrum**	**Width of the centrum**	**Length of the centrum**	**High of the neural arch**	**Total high**
**DBGI-54A**	100	180	100	-	-
**DBGI-37**	-	-	-	190	-
**DBGI-100 NA 2**	-	-	-	140	-
**DBGI-16**	120	135	80	-	480
**DBGI-181A**	175	150	110	-	-
**DBGI-19A**	-	-	-	140	-
**DBGI-24B**	155	130	130	-	-
**DBGI-100 NA 1**	-	-	-	190	-

**Caudal vertebrae**	**High of the centrum**	**Width of the centrum**	**Length of the centrum**	**High of the neural arch**	**Total high**
**DBGI-100V2**	190	230	45	-	-
**DBGI-192**	155	170	60	160	340
**DBGI-23B**	100	130	50	50?	210?
**DBGI-5B**	120	170	70	145	300
**DBGI-164**	110	210	110	-	-
**DBGI-34**	35	50	50	25	60

**DBGI-37B**	35	40	65	25	40
**DBGI-42A**	35	35	45	-	-
**DBGI-42B**	30	30	45	-	-
**DBGI-42C**	28	30	40	-	-
**DBGI-42D**	28	30	35	-	-
**DBGI-42E**	20	-	35	-	-
**DBGI-52A, 80 and 5C no measures**	-	-	-	-	-

**Chevrones**	Maximum length	**Maximum proximal width**	**Diaphisis width**
**Left Mc I**	200	105	65

**Manus**	Maximum length	**Maximum proximal width**	**Diaphisis width**
**Left Mc I**	200	105	65
**Left Mc II**	190	-	-
**Left Mc III**	190	-	-
**Left Mc IV**	160	70	45
**Left Mc V**	160	95	40
**I ungual**	100	60	-

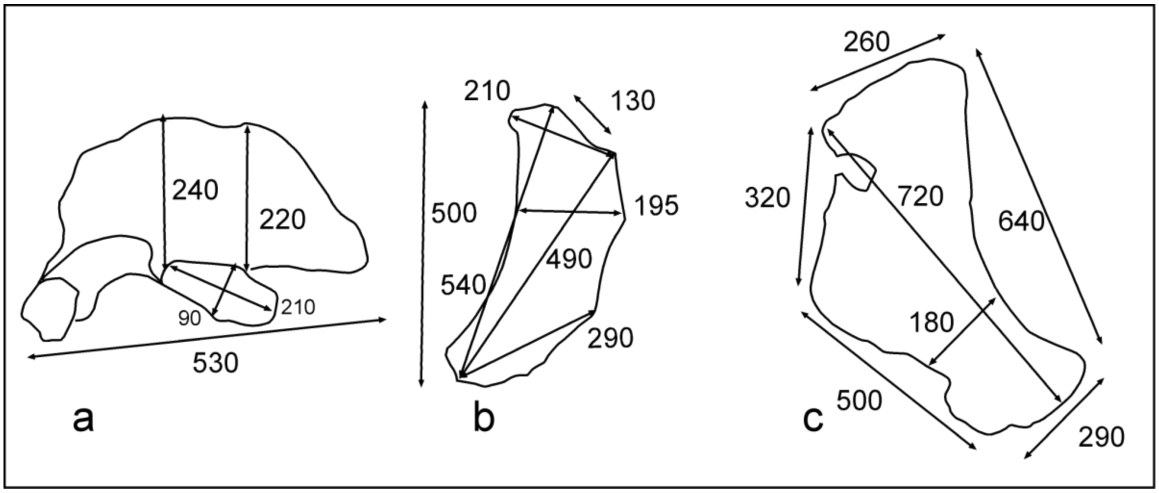

Fig. S6. Measurements (in mm) in the pelvic girdle of *Mierasaurus* gen. nov: a) left ilium (DBGI 100IL); b) right ischium (DBGI 69A), and c) right pubis (DBGI 195). Figure created by R.R.T. (© Fundación Conjunto Paleontológico de Teruel-Dinópolis) in Adobe Illustrator CS5 (www.adobe.com/es/products/illustrator.html).

VI. References

1. Stokes, W.L. Lower Cretaceous in Colorado Plateau. *American Association of Petroleum Geologists Bulletin* **36**, 1766-1776 (1952).
2. Kirkland, J.I. *et al.* Lower to Middle Cretaceous Dinosaur Faunas of the Central Colorado Plateau: A Key to Understanding 35 Million Years of Tectonics, Sedimentology, Evolution and Biogeography. *Brigham Young University Geology Studies*, **42** Part II, 69-103 (1997).
3. Kirkland, J.I., Suarez, M., Suarez, C. & Hunt-Foster, R. The Lower Cretaceous in east-central Utah: the Cedar Mountain Formation and its bounding strata. *Geology of the Intermountain West* **3**, 101-213 (2016).
4. Doelling, H.H. & Kuehne, P.A. Geologic map of the Short Canyon quadrangle, Emery County, Utah. *Utah Geological Survey Map* **255DM**, 1- 31 (2013).
5. Doelling, H.H & Kuehne, P.A. Geologic maps of the Klondike Bluffs, Mollie Hogans, and the Windows Section 7.5' quadrangles, Grand County, Utah, *Utah Geological Survey Map* **258DM**, Map **259DM**, Map **260DM**, 1-31 (2013).
6. Aubrey, W.M. A newly discovered, widespread fluvial facies and unconformity marking the Upper Jurassic/Lower Cretaceous boundary, Colorado Plateau. in The Upper Jurassic Morrison Formation—an interdisciplinary study, part I. (eds. Carpenter, K., Chure, D. & Kirkland, J.I.), *Modern Geology* **22**, 209-233 (1998).
7. Kirkland, J.I., *et al.* Distribution of Vertebrate faunas in the Cedar Mountain Formation, east-central Utah, in Vertebrate paleontology in Utah. (ed. Gillette, D.) *Utah Geological Survey Miscellaneous Publication* **99-1**, 201-217 (1999).
8. Senter P., Kirkland, J.I., Bird, J. & Bartlett, J.A. A new troodontid theropod dinosaur from the Lower Cretaceous of Utah. *PLoS One* **5** (12), e14329 (2010).
9. Kirkland, J.I., Zanno, L.E., Sampson, S.D., Clark, J.M. & DeBlieux, D.D. A primitive therizinosauroid dinosaur from the Early Cretaceous of Utah. *Nature* **435**, 84-87 (2005)
10. Zanno, L.E. The pectoral girdle and forelimb of the primitive therizinosauroid *Falcarius utahensis* (Theropoda, Maniraptora): Analyzing evolutionary trends within Therizinosauroidea. *Journal of Vertebrate Paleontology* **26**, 636-650 (2006).
11. Zanno, L.E. Osteology of *Falcarius utahensis* (Dinosauria: Theropoda) -characterizing the anatomy of basal therizinosaurs. *Zoological Journal of the Linnean Society* **158**, 196-230 (2010).

12. Senter P., Kirkland, J.I., DeBlieux, D.D., Madsen, S.K. & Toth, N. New dromaeosaurids (Dinosauria: Theropoda) from the Lower Cretaceous of Utah, and the evolution of the dromaeosaurid tail. *PLoS One* **7** (5), e36790 (2012).
13. McDonald, A.T. *et al.* New basal iguanodonts from the Cedar Mountain Formation of Utah and the evolution of thumb-spiked dinosaurs. *PloS One* **5** (11) e14075 (2010).
14. Kirkland, J.I. & Madsen, S.K. The Lower Cretaceous Cedar Mountain Formation, eastern Utah: The view up an always interesting learning curve. in *Field Guide to Geological excursions in southern Utah, Geological Society of America Rocky Mountain Section 2007 Annual Meeting.* (ed. Lund W.R.) *Utah Geological Association Publication* **35**, 1-108 (2007).
15. Kirkland, J.I., DeBlieux, D., Madsen, S.K. & Hunt, G.J. New dinosaurs from the base of the Cretaceous in eastern Utah suggest that the "so-called" basal Cretaceous calcrete in the Yellow Cat Member of the Cedar Mountain Formation, while not marking the Jurassic-Cretaceous unconformity, represents evolutionary time: *Journal of Vertebrate Paleontology Abstracts and Program*, 121-122 (2012).
16. Kirkland, J.I., Burge, D. & Gaston, R.A. Large dromaeosaurid (Theropoda) from the Lower Cretaceous of eastern Utah. *Hunteria* **2**, 1-16 (1993).
17. Kirkland, J.I. A polacanthid ankylosaur from the Early Cretaceous of eastern Utah, *in* Lower to Middle Cretaceous non-marine Cretaceous faunas. *New Mexico Museum of Natural History and Science Bulletin* **14**, 271-281 (1998).
18. Tidwell, V.C., Carpenter, K. & Brooks, W. New sauropod from the Lower Cretaceous of Utah, USA. *Oryctos* **2**, 21-37 (1999).
19. Kowallis, B.J., Britt, B.B., Greenhalgh, B.W. & Sprinkel, D.A. New U-Pb zircon ages from an ash bed in the Brushy Basin Member of the Morrison Formation near Hanksville, Utah. in Central Utah -diverse geology of a dynamic landscape. *Utah Geological Association Publication* **36**, 75-80 (2007).
20. Trujillo, K.C. & Kowallis, B.J. Recalibrated legacy $^{40}Ar/^{39}Ar$ ages for the Upper Jurassic Morrison Formation, Western Interior, U.S.A. *Geology of the Intermountain West* **2**, 1-8 (2015).
21. Ogg, J.G. & Hinnov L.A., Cretaceous. in *The geologic time scale*. (eds Grandstein, F.M., Ogg, J.G., Schmitz, M.D.& Ogg, G.M.), 793-853 (Elsevier 2012).
22. Cohen, K.M., Finney, S.C., Gibbard, P.L. & Fan, J.-X. The ICS International chronostratigraphic chart: updated. *Episodes* **36**, 199-204 (2013).

23. Greenhalgh, B.W. & Britt, B.B. Stratigraphy and sedimentology of the Morrison/Cedar Mountain formational boundary, east-central Utah. in *Central Utah—Diverse geology of a dynamic landscape.* (eds Willis, G.C., Hylland, M.D., Clark, D.L. & Chidsey, T.C, Jr.) *Utah Geological Association Publication* **36**, 81-100 (2007).

24. Britt, B.B., Eberth, D.A., Scheetz, R., Greenhalgh, B.W., & Stadtman, K.L. Taphonomy of debris-flow hosted dinosaur bonebeds at Dalton Wells, Utah (Lower Cretaceous, Cedar Mountain Formation, USA). *Palaeogeography, Palaeoclimatology, Palaeoecology* **280**, 1-22 (2009).

25. Ludvigson, G.A., *et al*. Correlation of Aptian-Albian carbon isotope excursions in continental strata of Cretaceous foreland basin of eastern Utah. *Journal of Sedimentary Research* **80**, 955-974 (2010).

26. Sames, B., Cifelli, R.L. & Schudack, M.E. The nonmarine Lower Cretaceous of the North American Western Interior foreland basin—new biostratigraphic results from ostracod correlations and early mammals, and their implications for paleontology and geology of the basin—an overview. *Earth Science Reviews* **101**, 207-224 (2010).

27. Sames, B. Early Cretaceous *Cypridea bosquet* 1852 in North America and Europe, in taxonomic studies in Early Cretaceous nonmarine Ostracoda of North America. (ed. Sames, B.) 345-431, Micropaleontology Press (2011).

28. Martin-Closa, C., Sames, B. & Schudack, M.E. Charophytes from the Upper Berriasian of the Western Interior basin of the United States. *Cretaceous Research* **46**, 11-23 (2013).

29. Hendrix, B., Moeller, A., Ludvigson, G.A., Joeckel, R.M. & Kirkland, J.I. A new approach to date paleosols in terrestrial strata: a case study using U-Pb zircon ages for the Yellow Cat Member of the Cedar Mountain Formation of eastern Utah. *Geological Society of American Abstracts with Programs* **47**, 597 (2015).

30. Zeigler, K.E. Stratigraphy, paleomagnetism and magnetostratigraphy of the Upper Triassic Chinle Group, north-central New Mexico and preliminary magnetostratigraphy of the Lower Cretaceous Cedar Mountain Formation, eastern Utah. (University of New Mexico, dissertation) 1-224 (2008).

31. Zeigler, K.E., Donohoo-Hurley, L., Kirkland, J. & Geissman, J. Preliminary paleomagnetic data from the Yellow Cat and Poison Strip Members of the Lower Cretaceous Cedar Mountain Formation (Barremian-Aptian), Green River, central Utah. *Geological Society of America Abstracts with* Programs **39**, 9 (2007).

32. Dodson, P. Counting dinosaurs -how many kinds were there? *Proceedings of the National Academy of Sciences USA* **87**, 7608-7612 (1990).

33. Wang S.C. & Dodson P. Estimating the diversity of dinosaurs. *Proceedings National Academy of Science USA* **103**, 13601-13605 (2006).

34. Brusatte S.L. Macroevolution and extinction. in *Dinosaur Paleobiology.* John Wiley & Sons, Ltd, p. 242-261 (2012).

35. Benson, R.B.J., *et al.* Rates of dinosaur body mass evolution indicate 170 million years of sustained ecological innovation on the avian stem lineage. *PLoS Biology* **12**, e1001853 (2014).

36. Starrfelt J. & Liow L.H. How many dinosaur species were there? -fossil bias and true richness estimated using a Poisson sampling model. *Philosophical Transactions Royal Society Series B* **371**, 20150219 (2016)

37. Britt, B.B., Scheetz, R.D., Whiting, M.F. & Wilhite, D.R. *Moabosaurus utahensis* n. gen., n. sp., a new sauropod from the Early Cretaceous (Aptian) of North America. *Contributions from the Museum of paleontology, University of Michigan* **32**, 189-243 (2017).

38. Toth, N.G. A preliminary taphonomic analysis of the Doelling's Bowl Bonebed—investigating the sedimentation and paleofauna from an Early Cretaceous site in east-central Utah. (Rapid City, South Dakota School of Mines and Technology, thesis) 1-79 (2010).

39. Martz, J.W. & Parker, W.G. Revised Lithostratigraphy of the Sonsela Member (Chinle Formation, Upper Triassic) in the southern part of Petrified Forest National Park, Arizona. *PLoS ONE* **5**, e9329 (2010).

40. Parker, W.G. & Martz, J.W. The Late Triassic (Norian) Adamanian–Revueltian tetrapod faunal transition in the Chinle Formation of Petrified Forest National Park, Arizona. *Earth and Environmental Science Transactions of the Royal Society of Edinburgh* **101**, 231-260 (2011).

41. Dries, S.G. *et al.* Stop five—micromorphology and paleoclimatologic significance of Mountain Lion Mesa "silcrete" and underlying paleosols. in *SEPM-NSF Workshop "Paleosols and Soil Surface Analog Systems"* (eds. Dries, S.G. *et al.*), 111-122 (Petrified Forest National Park, Arizona 2010).

42. Young, R.G. Dakota Group of Colorado Plateau. *American Association of Petroleum Geologists Bulletin* **44**, 158-194 (1960).

43. Carpenter, K. Where the sea meets the land -the unresolved Dakota problem in Utah. in Geology of Utah's far south (eds. MacLean, J.S., Biek, R F. & Huntoon, J.E.) Utah Geological Association Publication 43, 357-372 (2014).

44. Sprinkel, D.A., Madsen, S.K., Kirkland, J.I., Waanders, G.L. & Hunt, G.J. Cedar Mountain and Dakota Formations around Dinosaur National Monument—evidence of the first incursion of the Cretaceous Western Interior seaway into Utah. Utah Geological Survey Special Study 143, 1-21, (2012).
45. Carballido, J.L. & Sander, P.M. Postcranial axial skeleton of Europasaurus holgeri (Dinosauria, Sauropoda) from the Upper Jurassic of Germany: implications for sauropod ontogeny and phylogenetic relationships of basal Macronaria. Journal of Systematic Palaeontology 12, 335-87 (2014).
46. Goloboff, P.A., Farris J.S. & Nixon, K. TNT: tree analysis using new technology. Systematic Biology 54, 176-178 (2003)
47. Upchurch, P., Barrett, P.M. & Dodson, P. Sauropoda. in The Dinosauria: Second Edition (eds Weishampel, D.B., Dodson, P. & Osmólska, H.) 259-322 (University of California Press, 2004).
48. Mannion, P.D., Allain, R. & Moline, O. The earliest known titanosauriform sauropod dinosaur and the evolution of Brachiosauridae. Peerj 5:e3217 (2017).
49. Royo-Torres, R., Cobos, A. & Alcalá, L. A giant European dinosaur and a new sauropod clade. Science 314, 1925-1927 (2006).
50. Monbaron, M., Russell, D.A. & Taquet, P. Atlasaurus imelakei n.g., n.sp., a brachiosaurid-like sauropod from the Middle Jurassic of Morocco. Comptes Rendus de l'Académie des Sciences à Paris, Sciences de la Terre et des Planètes 329, 519-526 (1999).
